# Estimating PM_2.5_ speciation concentrations using prototype 4.4 km-resolution MISR aerosol properties over Southern California

**DOI:** 10.1016/j.atmosenv.2018.03.019

**Published:** 2018-03-10

**Authors:** Xia Meng, Michael J. Garay, David J. Diner, Olga V. Kalashnikova, Jin Xu, Yang Liu

**Affiliations:** a Department of Environmental Health, Rollins School of Public Health, Emory University, Atlanta, GA, United States; b Jet Propulsion Laboratory, California Institute of Technology, Pasadena, CA, United States; c California Air Resources Board, Sacramento, CA, United States

**Keywords:** PM_2.5_ speciation, MISR fractional AOD

## Abstract

Research efforts to better characterize the differential toxicity of PM_2.5_ (particles with aerodynamic diameters less than or equal to 2.5 μm) speciation are often hindered by the sparse or non-existent coverage of ground monitors. The Multi-angle Imaging SpectroRadiometer (MISR) aboard NASA’s Terra satellite is one of few satellite aerosol sensors providing information of aerosol shape, size and extinction globally for a long and continuous period that can be used to estimate PM_2.5_ speciation concentrations since year 2000. Currently, MISR only provides a 17.6 km product for its entire mission with global coverage every 9 days, a bit too coarse for air pollution health effects research and to capture local spatial variability of PM_2.5_ speciation. In this study, generalized additive models (GAMs) were developed using MISR prototype 4.4 km-resolution aerosol data with meteorological variables and geographical indicators, to predict ground-level concentrations of PM_2.5_ sulfate, nitrate, organic carbon (OC) and elemental carbon (EC) in Southern California between 2001 and 2015 at the daily level. The GAMs are able to explain 66%, 62%, 55% and 58% of the daily variability in PM_2.5_ sulfate, nitrate, OC and EC concentrations during the whole study period, respectively. Predicted concentrations capture large regional patterns as well as fine gradients of the four PM_2.5_ species in urban areas of Los Angeles and other counties, as well as in the Central Valley. This study is the first attempt to use MISR prototype 4.4 km-resolution AOD (aerosol optical depth) components data to predict PM_2.5_ sulfate, nitrate, OC and EC concentrations at the sub-regional scale. In spite of its low temporal sampling frequency, our analysis suggests that the MISR 4.4 km fractional AODs provide a promising way to capture the spatial hotspots and long-term temporal trends of PM_2.5_ speciation, understand the effectiveness of air quality controls, and allow our estimated PM_2.5_ speciation data to be linked with common spatial units such as census tract or zip code in epidemiological studies. This modeling strategy needs to be validated in other regions when more MISR 4.4 km data becoming available in the future.

## GRAPHICAL ABSTRACT

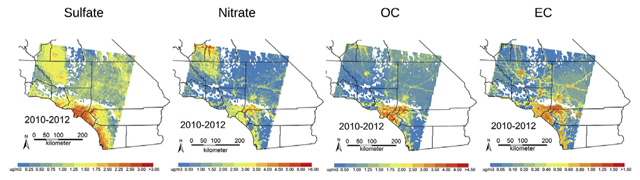

## 1. Introduction

Ambient fine particulate matter (PM_2.5,_ airborne particles with aerodynamic diameter less than 2.5 μm) has been proven to be a major environmental risk factor to public health (Dominici et al., 2006; Franklin et al., 2007; Pope et al., 2002; Raaschou-Nielsen et al., 2013; Turner et al., 2011). In 2013, there were an estimated 2.9 million deaths (5.3% of all global deaths) and 69.7 million Disability-Adjusted Life Years (DALYs) caused by outdoor PM_2.5_ air pollution globally (Forouzanfar et al., 2015). Recently, an increasing number of studies highlight the adverse health effects of PM_2.5_ components, including sulfate, nitrate, organic carbon (OC), elemental carbon (EC), as well as some trace elements (Cao et al., 2012; Peng et al., 2009; Thurston et al., 2016; Zanobetti et al., 2008). The chemical composition of PM_2.5_ is complex and varies in time and space due to different emission sources and the subsequent chemical reactions, physical interactions, and geographic transport. Understanding the temporal and spatial characteristics of PM_2.5_ speciation is one of the key steps to identify emission sources, provide support of exposure assessment for health effects studies, and ultimately support the making of effective air pollution control policies and protecting public health. Research efforts to better characterize the differential toxicity of PM_2.5_ speciation are often hindered by the sparse or non-existent coverage of ground monitors. The United States has built the most extensive ground PM_2.5_ speciation monitoring network with 300 + stations nationwide, including the National PM_2.5_ Chemical Speciation Network (CSN) and the Inter-agency Monitoring of Protected Visual Environments (IMPROVE) Network (Solomon et al., 2014). Nonetheless, these networks are still sparse compared to networks monitoring total PM_2.5_ mass concentrations, and unable to fully capture the spatial pattern of PM_2.5_ speciation within a city, where the spatial variability of PM_2.5_ level is high. Moreover, many areas have no monitoring sites. Hence, estimating PM_2.5_ speciation with models plays a significant role in understanding PM_2.5_ speciation levels and distributions.

Chemical transport models (CTMs) such as the Goddard Earth Observing System (GEOS)-Chem and the Community Multi-scale Air Quality (CMAQ) are able to help predict temporal-spatial trends of PM_2.5_ speciation since the simulations do not rely on ground measurements of PM_2.5_ speciation. A CTM is a physically- and chemically-derived three-dimensional model that simulates PM_2.5_ speciation concentrations using inputs such as emission source data, meteorological data, particle physical and chemical properties, and receptor data. In general, however, the simulated concentrations of PM_2.5_ and its components from CTMs often have relatively high uncertainty (Baek et al., 2011; Kim et al., 2015; Mebust et al., 2003; Smyth et al., 2009). For example, the results from Yahya et al. (2014) showed that correlation coefficients between daily measurements and modelled concentrations based on Weather Research and Forecasting (WRF)-Chem with a spatial resolution of 12 km ranged from 0.2 to 0.6 for PM_2.5_ sulfate, from 0.1 to0.7 for PM_2.5_ nitrate, from 0.1 to 0.4 for PM_2.5_ OC and from 0.2 to 0.7 for PM_2.5_ EC in winter (December–February) and the ozone season (May–September) during 2009–2011 (Yahya et al., 2014). Walker et al. (2012) reported that the normalized mean bias (NMB) between annual mean measurements and modelled concentrations based on GEOS-Chem with spatial resolution of 0.5° × 0.667° were 3%, −62% and −38% for sulfate, nitrate and ammonium in California in 2009, respectively (Walker et al., 2012). Another study using a regional air quality model developed by University of California at Davis/California Institute of Technology (UCD/CIT) showed that the simulated concentrations of PM_2.5_ species were lower than measured concentrations, ranging from 12% to 64%, from 16% to 60% and from 18% to 73% for EC, OC and SNA (sulfate, nitrate and ammonium) respectively at six ground monitoring sites in California based on the 7-year averaged data of 2000–2006 (Mahmud et al., 2010). Another WRF-Chem simulation at 4-km resolution with improved emissions was conducted in San Joaquin Valley of California during October 2012 to September 2013. Comparing with ground observations at Fresno, CA, the predicted concentrations were biased by −6%, +75%, −63%, and +4% in the cold season and by −41%, −17%, −65%, and +53% in the warm season for PM_2.5_ sulfate, nitrate, organic matter (OM), and EC, respectively (Wu et al., 2017).

Another approach to predict ground-level particulate pollution is based on remotely sensed aerosol products. Although statistical models driven by remotely sensed total column aerosol optical depth (AOD) can effectively predict ground-level total PM_2.5_ mass concentrations with wide spatial and temporal coverages (Hu et al., 2014; Lee et al., 2011; Ma et al., 2016), most satellite aerosol products cannot be used for estimating PM_2.5_ speciation because they are unable to distinguish the physical and chemical characteristics of aerosols. With its unique multiangle design, the Multiangle Imaging SpectroRadiometer (MISR) aboard NASA’s Terra spacecraft provides information of aerosol shape, size and extinction globally. Detailed descriptions of the MISR retrieval algorithm and MISR data structure are given elsewhere (Liu et al., 2007a; Martonchik et al., 2009). Briefly, MISR pre-selects a set of aerosol mixtures that consist of several aerosol components, which are defined by the particle size distribution, shape, complex index of refraction, and scale height. The retrieval ranks the mixtures by their agreement with MISR’s multiangle observations within a specific region, and uses spatial information to account for the underlying surface as well as spectral similarity constraints on the angular distribution of reflected radiation to help select the models. MISR orbits the Earth nearly 15 times per day with a swath width of 380 km and each path is repeated every 16 days, which allows MISR to view the entire Earth between ± 82° latitude in nine days, with more frequent coverage at the higher latitudes. MISR’s low sampling frequency makes it unsuitable for daily air quality monitoring or short-term epidemiological studies, such as time series studies on the associations between health outcomes and daily PM_2.5_ speciation concentrations. However, the rich aerosol information and broad spatial coverage enable it to be used for studying long-term spatiotemporal trends of PM_2.5_ speciation and health effects related to long-term exposures of PM_2.5_ components. Although MISR-retrieved aerosol microphysical properties do not directly correspond to chemical composition, previous research has shown potential to use such information in predicting ground-level PM_2.5_ speciation (Liu et al. 2007a, 2007b). For example, MISR fractional AODs at 17.6 km resolution have been used to predict fine particle sulfate concentrations in the US (Liu et al., 2009). This resolution is a bit too coarse for air pollution health effects research and to capture local hotspots and high spatial variability of PM_2.5_ speciation. In order to reflect finer spatial details of PM_2.5_ speciation, the MISR project is nearing completion of a 4.4 km global reprocessing (version 23), which is expected to be available in mid-2018. A prototype version of this product has demonstrated improved quality in retrieved total AOD in comparison with measurements from the Aerosol Robotic Network (AERONET) (Garay et al., 2017). Moreover, the assessment by Garay el al. 2017 shows that the MISR 4.4-km algorithm reduces the underestimation at high AOD levels and increases the spatial sampling coverage of AOD compared with the17.6-km data. The finer spatial resolution of this product is also better able to resolve spatial gradients in AOD as shown in a number of comparison analyses. Franklin et al. used total and size-fractionated AOD (AOD small, AOD medium and AOD large) from MISR Local Mode data at 4.4 km resolution to generate prediction maps of PM_2.5_ and PM_10_ over Southern California (Franklin et al., 2017). They did not apply MISR mixtures and fractional AODs in modeling, which represent not only the aerosol size but also aerosol shape and extinction properties, and prediction maps of PM_2.5_ species were not produced in their study domain. Another issue with regard to the study of Franklin et al. (2017) is that the MISR Local Mode data is limited to pre-defined target areas (https://misr.jpl.nasa.gov/getData/localMode/). Further research is needed to evaluate whether the upcoming globally available MISR 4.4 km operational data can enable worldwide characterization of PM_2.5_ hotspots and detect the spatial gradients of PM_2.5_ constituents within urban areas at a higher spatial resolution.

The objectives of this study are to develop statistical models with prototype MISR 4.4-km global mode aerosol microphysical properties to predict ground-level concentrations of major PM_2.5_ chemical components, including sulfate, nitrate, OC and EC in Southern California, and explore the long-term temporal and spatial trends of these PM_2.5_ components based on model predictions. This case study illustrates a novel capability for predicting concentrations and capturing the high spatial variability of PM_2.5_ species using satellite remote sensing data at relatively high spatial resolution and provide references for future applications, e.g. epidemiological studies that focus on PM_2.5_ differential toxicity requiring exposure estimates of a high spatial resolution, spatial analysis to detect hotspots of certain PM_2.5_ constituents, and long-term trends analysis of PM_2.5_ speciation for assessing emission control policies. Once the Version 23 MISR aerosol product is publicly released and the global data record reprocessed to 4.4-km resolution, the methodology can be extended to other regions of the world.

## 2. Data and methods

### 2.1. Study area

**Fig. 1.**
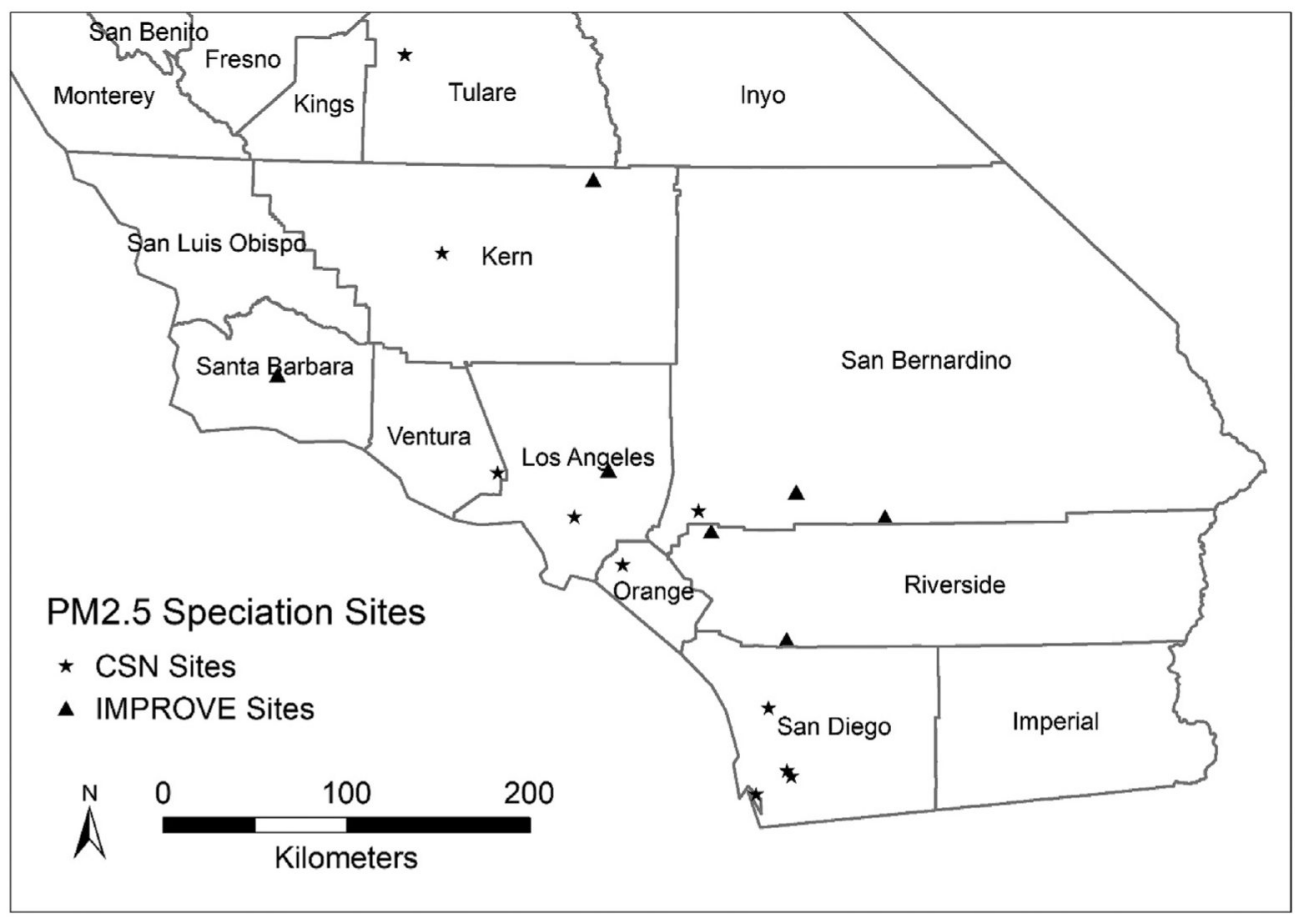
Locations of PM_2.5_ speciation monitoring sites from CSN and IMPROVE networks in Southern California. Counties covered by the MISR aerosol data of blocks 62–64 in path 41 include Kern, Santa Barbara, Ventura, Los Angeles, Orange, and part of Fresno, Kings, Tulare, Inyo, San Luis Obispo, San Bernardino, Riverside and San Diego County.

Our study domain is set in Southern California, including the counties of Kern, Santa Barbara, Ventura, Los Angeles, Orange, and part of the counties of Fresno, Kings, Tulare, Inyo, San Luis Obispo, San Bernardino, Riverside and San Diego. This area is covered by the MISR aerosol data of blocks 62–64 in path 41 (Fig. 1).

### 2.2. Data

#### 2.2.1. MISR prototype 4.4-km resolution data

This analysis used prototype of the MISR Level 2 (L2) aerosol data for the years 2001–2015, which was provided by the Jet Propulsion Laboratory (JPL). Differences between this prototype product and the data that will be delivered as the new operational (V23) aerosol product are minor and will not affect the conclusions of this work. The spatial resolution of the aerosol product is 4.4 km × 4.4 km. According to the latest validation results, the correlation coefficient between MISR4.4 km AOD and AERONET AOD is 0.96 based on data of AERONET-DRAGON (Distributed Regional Aerosol Gridded Observations Network) sites in San Joaquin Valley, Washington DC-Baltimore, Seoul of Korea, and Osaka of Japan (Garay et al., 2017). This aerosol dataset used in this study is of blocks 62–64 in path 41, and make use of the same 74 aerosol mixtures as in the operational (V22) product. The 74 mixtures are constructed from eight fractional AOD components (AOD1, AOD 2, AOD 3, AOD 6, AOD 8, AOD 14, AOD 19 and AOD 21 in the MISR Aerosol Physical and Optical Properties database) that represent different particle characteristics of size, shape and complex refractive index. The eight fractional AOD components (listed in Table S1) are named starting with particle shape, followed by a qualitative scattering property designation and ending with the effective radius for a number-weighted log-normal distribution. Single scattering albedo at 558 nm wavelength is added when necessary to distinguish components.

The fractional AOD values can be calculated for any given MISR aerosol observation from equation (1) (Liu et al. 2007a, 2009):

(1) $${\text{Fractional AOD}\;}_{\text{i}(\text{i}=1-8)}\;=\;\frac{\sum_{j=1}^{74}\alpha AOD_{\text{mixture}\;\text{j}}\;\times \;\;Fraction_{\text{component i in mixture j}}}{No.\;\;of\;successful\;mixtures}$$

where AOD_mixture j_ is the AOD of mixture j; Fraction_component i in mixture j_ is the contribution of component i to the total AOD for mixture j; if mixture j is retrieved successfully, then α = 1, otherwise α = 0.

#### 2.2.2. PM_2.5_ speciation data

Daily-averaged PM_2.5_ mass and speciation concentrations from the U.S. Environmental Protection Agency’s (EPA) urban Chemical Speciation Network (CSN) (Solomon et al., 2014) and remote/rural Interagency Monitoring of Protected Visual Environments (IMPROVE) network (Malm et al., 1994) were obtained from EPA’s Air Quality System (AQS) (http://aqsdr1.epa.gov) and Federal Land Manager Environmental Database (http://views.cira.colostate.edu/fed), respectively. The locations of PM_2.5_ speciation sites in our study domain are displayed in Fig. 1. Sulfate (SO_4_^2−^), nitrate (NO_3_^−^), organic carbon (OC) and elemental carbon (EC) were selected for model development, since they were the major PM_2.5_ components in the study domain (CARB, 2016). The sampling frequency is 1-in-3 or 1-in-6 days for CSN and 1-in-3 days for IMPROVE. The sampling and analytical methods of sulfate and nitrate from CSN and IMPROVE are only slightly different (Solomon et al., 2014); and the comparison shows high consistency of measurements from the two monitoring networks for nitrate and sul-fate, respectively (Hand et al., 2011, 2012). Therefore, no corrections were done for sulfate and nitrate from different networks. EC and OC PM_2.5_ data from CSN were adjusted in order to be comparable with those from IMPROVE. The differences in the IMPROVE and CSN carbon measurements are mainly caused by different samplers and analytical methods. The details about the differences were discussed elsewhere (Solomon et al., 2014) and were summarized in supplementary material in this paper. For EC and OC data from CSN, blank values were subtracted from the data if they were measured by Thermal Optical Re-flectance (TOR) method; if they were measured by Thermal Optical Transmittance (TOT) method, the EC data was adjusted by multiplying by 1.3 to match the EC data from IMPROVE, while the OC data was converted with the following (equation 2) (Malm et al., 2011):

(2) $$\text{OC\_adjust}\;=\;\left(\left({\text{OC}}_{\text{CSN}}-0.3\;\times \;{\text{EC}}_{\text{CSN}}\right)-\text{A}\right)/\text{M}$$

The values of parameters A and M were listed in Table S2. The equation was built based on data of PM_2.5_ OC and EC concentrations from collocated sites of CSN and IMPROVE in 2005 and 2006. A represented the monthly positive organic artifact resulting from filter adsorption of semivolatile organic compound (SVOC), and M indicated the multiplicative negative organic carbon artifact related with volatilization of collected OC mass (Malm et al., 2011).

#### 2.2.3. Meteorological and land use predictors

Previous research showed that elevation and seasonal variability of PBLH might contribute significantly to the differences of PM_2.5_ nitrate and OC in central and southern California in winter (Hand et al., 2014). Daily mean planetary boundary layer height (PBLH) data with 32 km spatial resolution were obtained from the North American Regional Reanalysis (NARR) data products (Mesinger et al., 2006). Additionally, variables indicating local surface cover, geographic topography and traffic emission, which could reflect the influence of local emissions and environment to concentrations of PM_2.5_ species, were included in this study. Moderate Resolution Imaging Spectroradiometer (MODIS) collection 6 MOD13A2 (https://modis.gsfc.nasa.gov/data/dataprod/mod13.php) normalized difference vegetation index (NDVI) were used in this study to indicate the changes of land surface cover. This NDVI product is provided every 16 days at 1-km spatial resolution as a gridded Level-3 product in the Sinusoidal projection. Digital maps of major roads (including highways) for year 2011 were obtained from ESRI StreetMap USA (Environmental Systems Research Institute, Inc., Redlands, CA) to indicate the local traffic emission. Elevation data were extracted from the prototype 4.4 km MISR aerosol dataset that were based on the Digital Elevation Model (DEM) (https://asterweb.jpl.nasa.gov/gdem.asp) used by the MISR project, which indicates the geographic topography. Elevation data remain constant for the entire study periods.

#### 2.2.4. Spatial and temporal indicators

The geographic coordinates of CSN and IMPROVE sites (x, y) were included as spatial indicators to reflect the potential impact of site locations on the association between PM_2.5_ speciation and MISR fractional AODs. Day of the year (DOY) and the year index (Year) were included as covariates to account for systematic seasonal and long-term trends.

### 2.3. Data integration

A 2.5 km-radius buffer was made for each CSN and IMPROVE site so that the buffer area is approximately equal to the area of a MISR 4.4 km pixel. MISR fractional AODs were assigned to each CSN and IMPROVE site if the MISR pixel centroid falls within the buffer zone. The average NDVI of all matched pixels in the buffer zone was assigned to the site. Inverse-distance weighting was used to interpolate the PBLH values to the locations of CSN and IMPROVE sites. Lengths of major roads within the buffer zone were calculated for each CSN and IMPROVE site. All covariates of the prediction dataset were integrated based on the locations of the MISR 4.4 km grid cells.

### 2.4. Model development

Generalized additive models (GAM) were developed for each PM_2.5_ component with MISR fractional AODs as primary predictors and other variables, including PBLH, land use predictors, temporal indicators and spatial indicators mentioned above. A GAM allows the predictor variables to have nonlinear relationships with the dependent variable by using semi-parametric spline smoothers. The model structure for sulfate can be expressed in equation (3) as follows and the model structures of nitrate, OC and EC were similar to that of sulfate:

(3) $${\text{PM}}_{2.5}\;\text{sulfate}\;=\sum_{i=1}^{8}f_{i}\left(MISR\;fractional\;AOD_{i}\right)+f(\text{DOY})+f(\text{Year})\;+\;f_{x,y}(\text{x},\text{y})+f(\;\text{elevation}\;)+f(\text{NDVI})+f(\text{road length})+f(\text{PBLH})$$

where *f*_*i*_ (MISR fractional AOD_i_) is smooth term of MISR fractional AOD_i_; *f*_*x,y*_ (x,y) is a 2-D smooth spatial function of site locations; *f* (DOY) and *f* (Year) are a nonlinear term of DOY and the year index varying smoothly in time, respectively; and *f* (elevation), *f* (NDVI), *f* (road length) and *f* (PBLH)are smooth terms of elevation, NDVI, road length and PBLH, respectively. Cubic regression splines are used for all smooth terms except *f*_*x,y*_ (x,y).

Predictor variables statistically significant at the α = 0.10 level were kept in the final model. Since MISR data are sparse and the data size is small in this analysis, adjusted coefficient of determination (R^2^) was used as the selection criterion and as a primary indicator of model performance (Liu et al., 2007a). Leave-one-out-cross-validation (LOOCV) was applied to estimate model prediction accuracy and to test for potential model overfitting. Cook’s Distance was used to identify influential data points deviating significantly from the overall trend of the dataset of measured and predicted values. The Cook’s Distance values were compared to the F distribution with p+1 degrees of freedom in the numerator and *N*-p-1 degree of freedom in the denominator (p is the number of predictor variables in the GAM, and N is the total number of the data used for model development) (Liu et al., 2007a). If the Cook’s Distance value of a data point is greater than the 20th percentile value of this F distribution, the data point would be removed from the dataset and the model would be regenerated. Root-mean-square errors (RMSE, defined as standard deviation of model residuals), absolute prediction errors (APE, defined as absolute values of model residuals) and normalized mean errors (NME, defined as mean of APE divide by mean of measured concentrations of PM_2.5_ species) were calculated to quantify model prediction errors.

## 3. Results and discussion

### 3.1. Descriptive statistics of the model fitting dataset

After matching ground measurements of PM_2.5_ species with MISR fractional AODs and other predictor variables, we obtained 622 site-days of data for the years 2001–2015 for sulfate and nitrate, and 624 site-days of data for the years 2001–2015 for OC and EC in the model fitting datasets. All successfully retrieved AOD values were involved in model development. In the model fitting dataset, mean values of daily PM_2.5_ sulfate, nitrate, OC and EC are 1.63 μg/m^3^, 2.42 μg/m^3^, 2.12 μg/m^3^, and 0.59 μg/m^3^, respectively, accounting for 17%, 21%, 21% and 6% of average daily PM_2.5_ mass concentrations, respectively. This suggests that secondary inorganic mass and organic mass are the major constituents in PM_2.5_ in our study domain.

### 3.2. Results of model fitting and cross-validation

Table 1 summarizes the model fitting results for PM_2.5_ speciation. The adjusted R^2^ values of final models vary from 0.55 to 0.66 with the highest value for the sulfate model and the lowest value for the OC model, indicating that the GAMs developed in this study are able to capture majority of variability for the four PM_2.5_ species. The LOOCV R^2^ values decrease slightly by 0.04–0.06 compared to the model adjusted R^2^ values, showing high stability and little overfitting of the models. Comparing with the work of Franklin et al., (2017), we used the MISR fractional AODs in modeling, which represents not only aerosol size but also aerosol shape and extinct properties. The model performances in this study are more stable. In the previous work, the cross validation R^2^ values drop down to 0.14, 0.69 and 0.39 from 0.44, 0.74 and 0.72 for PM_2.5_ OC, sulfate and nitrate, respectively, indicating significant model overfitting for OC and nitrate likely due to a small sample size (Franklin et al., 2017). The unexplained variability of PM_2.5_ species in this study may be attributed to that 1) the MISR fractional AODs are not designed to specifically match PM_2.5_ speciation, so they are unable to fully represent the physical and chemical properties of PM_2.5_ components; 2) we use column fractional AODs to predict ground level PM_2.5_ species without accounting for the vertical profile of aerosols, since the study domain is relatively small and the aerosol vertical profile data for the long study period and fine spatial resolution for this analysis was not available. Instead, we included PBLH as a covariate in models to partially reflect the effect of aerosol vertical profile on ground-level PM_2.5_ species concentrations; 3) other known and unknown indicators related to concentrations of PM_2.5_ species, such as industrial emissions, are not included in model development, which might also limit the capability of the models to predict PM_2.5_ species variability.

MISR fractional AODs are significant predictors in all models. MISR AOD2, AOD3 and AOD19 are the most frequently used AOD components, probably because their sizes are highly consistent with that of PM_2.5_. EC is a strong light absorbing PM_2.5_ component; therefore MISR light-absorbing AOD14 with a single scattering albedo of 0.80 at 558 nm contributes significantly to the EC model as expected. However, MISR AOD2 representing non-absorbing aerosol is also positively related with PM_2.5_ EC concentrations in the model, which might be because MISR AOD14 itself is not sensitive enough to predict the variability of EC concentrations and MISR AOD2 could provide supplementary information given that the size distributions of AOD2 and AOD14 are identical. Two non-absorbing components AOD2 and AOD3 are involved in the OC model. OC may contain light-absorbing brown carbon whose absorption is stronger at near-ultraviolet and blue wavelengths and increases steeply towards shorter wavelengths (Andreae and Gelencser, 2006; Sun et al., 2007). It is difficult for MISR fractional AODs to be sensitive to light-absorbing OC since MISR doesn’t have ultraviolet spectral band. The spatial and temporal indicators are involved in all of the four final models, reflecting the spatial distribution, seasonal pattern and long-term time series trend of the four PM_2.5_ species.

The smooth terms of the predictor variables in the fitted models of the four PM_2.5_ species are displayed in Figure S1. MISR fractional AODs as smooth functions of PM_2.5_ species concentrations with monotonically decreasing trends are removed from the model since it cannot be interpreted physically. Most of the MISR AOD components in the models show nonlinear relationships with PM_2.5_ speciation, as MISR AOD components are not defined to match the PM_2.5_ speciation exactly. The standard error curves (dashed lines) become nonconvergent at high values of MISR AOD, where fewer data support the model fitting and the estimates are less stable. PBLH shows a negative association with PM_2.5_ speciation that reflects the effect of vertical distribution on surface PM_2.5_ concentrations. Lengths of major roads are positively associated with concentrations of PM_2.5_ nitrate, OC and EC, reflecting the contribution of traffic emissions to PM_2.5_ nitrate, OC and EC.

**Fig. 2.**
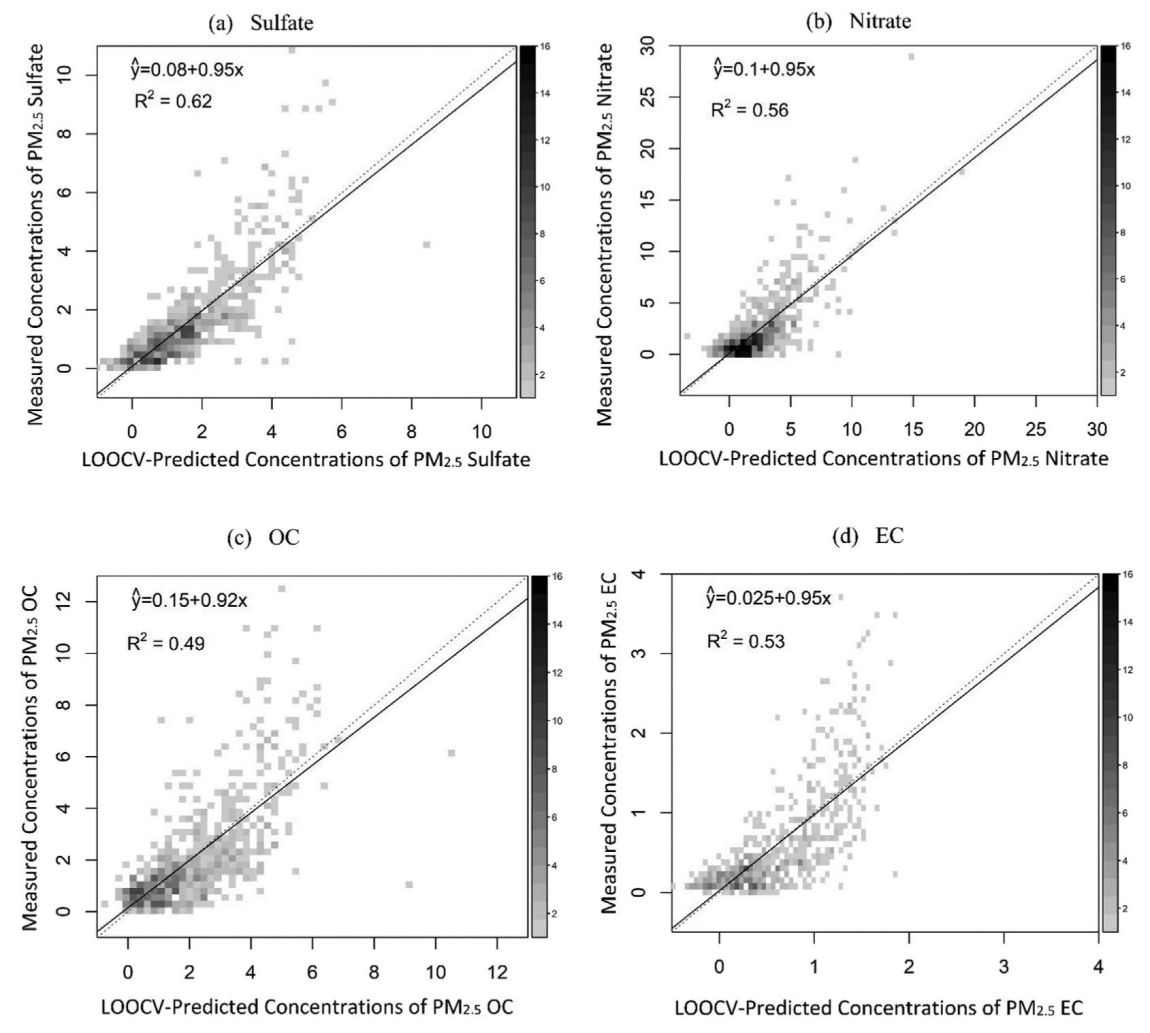
Linear regressions between measured and predicted concentrations of PM_2.5_ (a) sulfate, (b) nitrate, (c) OC and (d) EC. The y axis represents measurements of PM_2.5_ speciation with unit of μg/m^3^; the x axis represents prediction of PM_2.5_ speciation from LOOCV with unit of μg/m^3^. Dash line is the 1:1 line, and solid line is simple linear regression line between measurements and predictions. The gradient legend bar in the left of each plot represents the count of data included in each square in the plot.

Fig. 2 shows the comparisons of observed and model-predicted concentrations from LOOCV. Most of the predictions are linearly correlated with corresponding measured values, and the sulfate model shows the best linear fit. The models have greater uncertainties with the ground measurements of PM_2.5_ speciation at low pollution levels, where measurement errors may bias the association between PM_2.5_ speciation and MISR fractional AODs. Additionally, most measurements of low PM_2.5_ species concentrations are from IMPROVE monitors located in National Parks or rural areas, where the PM_2.5_ levels and variability are relatively small and difficult to predict using regression models. Additionally, the AOD retrieval error is another contributor to prediction uncertainty (Hoff and Christopher, 2009). Moreover, the AOD values at these pollution levels tend to be small, (e.g. less than 0.15 or even less than 0.05). Previous studies suggested that MISR retrieval errors of aerosol microphysical information are higher at lower AOD values (Liu et al., 2009), and such errors may lead to higher prediction errors at lower concentrations of PM_2.5_ species. At high pollution levels, the models tend to underestimate the PM_2.5_ species concentrations, most likely due to the small sample size at high aerosol loadings, which limits models capability to establish robust associations between high PM_2.5_ species concentrations and MISR fractional AODs and other predictor variables. The underestimation could be reduced when more MISR AOD data and more predictor variables such as daily traffic volume or emissions data with a high temporal resolution that could reflect daily high levels of PM_2.5_ speciation become available.

The APE, RMSE and NME values of measured and model predicted concentrations of PM_2.5_ sulfate, nitrate, OC and EC are shown in Table 2. The NME values are 37%, 54%, 45%, and 51% for predicting daily PM_2.5_ sulfate, nitrate, OC and EC, respectively. The uncertainty at low concentrations of PM_2.5_ species contributes to the majority of the prediction errors. Comparing to CSN sites, the prediction errors are higher in IMPROVE sites where the PM_2.5_ levels are much lower (Table S3). Taking EC for example, the NME values calculated based on daily measured and model predicted concentrations are 39% and 66% in CSN sites and IMPROVE sites, respectively; meanwhile, the EC measured concentrations in CSN sites are 2.6 times the levels in IMPROVE sites. The underestimation at high concentrations of PM_2.5_ species is another reason contributing to the prediction error, which is reflected in Fig. 2.

The results of model fitting demonstrate that the models predicting concentrations of PM_2.5_ sulfate and nitrate perform better than predicting PM_2.5_ carbonaceous particles. In particular, the PM_2.5_ sulfate model shows the best capacity in terms of accuracy and stability. Additionally, the model adjusted R^2^ and LOOCV R^2^ decrease significantly if removing fractional AODs from the GAMs for sulfate and nitrate, while the changes are slight for OC and EC, indicating that MISR fractional AODs contribute more information for predicting sulfate and nitrate than predicting carbonaceous species. For example, the LOOCV R^2^ decrease by 0.11 and 0.29 for sulfate and nitrate, respectively, and by 0.01 for both OC and EC. For one reason, the composition and microphysical properties are relatively simple and well documented for sulfate and nitrate but more variable and less known for OC, therefore, it is difficult for MISR algorithm to distinguish light absorbing mixtures from non-light-absorbing mixtures (Liu et al., 2007b). Moreover, low levels of EC concentrations and lack of properly defined light-absorbing AOD components related with EC in the MISR particle dataset also likely negatively affect model performance with respect to EC.

### 3.3. Spatial and temporal trends of predictions for PM_2.5_ sulfate, nitrate, OC and EC

**Fig. 3.**
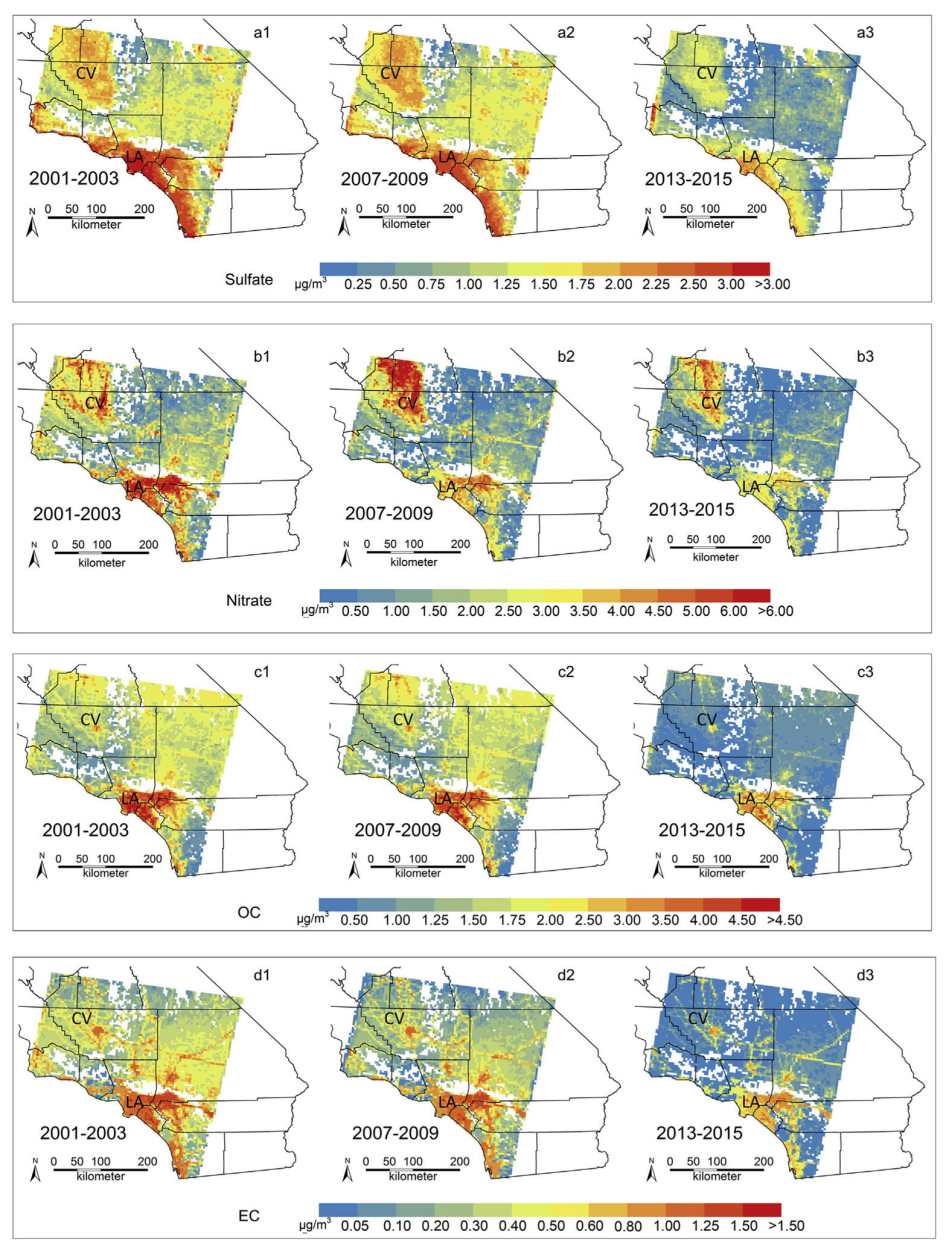
Prediction maps of 3-year averaged annual mean concentrations of ground-level PM_2.5_ sulfate, nitrate, OC and EC based on GAMs from days when the corresponding MISR AOD values of 4.4-km resolution were available. Character a, b, c, and d in the figures denote prediction maps for sulfate, nitrate, OC and EC, respectively. Numbers 1–3 in the figures denote 3-year averaged annual mean concentrations maps for years 2001–2003, 2007–2009 and 2013–2015, respectively. LA means Los Angeles urban area; CV means Central Valley area.

Fig. 3 shows the spatial distributions of mean annual concentrations of PM_2.5_ sulfate, nitrate, OC and EC predictions for every three years. As indicators of high population levels and locations of major cities, Figure S2 in the supplementary material shows (a) locations of ports, (b) a map of major roads and (c) distribution of nighttime city light assembled from data acquired by the Suomi National Polar-orbiting Partnership (NPP) satellite (Cao et al., 2014) in April and October 2012. In general, the concentrations of the four PM_2.5_ components have decreased gradually during the study period, especially after 2010, and remained relatively flat since then. Higher levels of the four PM_2.5_ components are seen mainly in the Central Valley, Los Angeles and other urban areas. There are several major ports located along the coastline of Southern California (see Figure S2 (a)), in particular, the Ports of Los Angeles and Long Beach comprise the largest port complex in the United States and handle one-fourth of all container cargo traffic in the United States (http://www.dot.ca.gov/). These areas along the coastline with seaports have the highest PM_2.5_ sulfate concentration, particularly in Los Angeles area, indicating the contribution of ship emissions on sulfate pollution in Southern California. For nitrate, higher concentrations appear at areas with intensive road network and the Central Valley, suggesting that traffic and agricultural activities might be the major sources of nitrate precursor emissions. The hotspots of PM_2.5_ carbonaceous components appear in urban areas and along major roads, showing the potential influence of anthropogenic combustion and traffic emissions. There are some negative but near-zero predicted values of PM_2.5_ species after 2010 appearing in mountain and rural wilderness areas where particulate pollutant levels are too low to be predicted accurately with our models; however, population density is also low in those areas. Therefore, uncertainties in predicting the low concentrations of PM_2.5_ species may have minor influence on future policy assessment and health studies.

Comparing with the previous study using the 17.6 km-resolution MISR aerosol product to predict ground-level concentrations of PM_2.5_ sulfate (Liu et al., 2009), the high spatial resolution of the prototype MISR 4.4 km-resolution fractional AODs enables our approach to identify hotspots and small concentration gradients of PM_2.5_ species at the city scale, and therefore, allows our estimated PM_2.5_ speciation data to be linked with common spatial units such as census tract or zip code in epidemiological studies on long-term exposure of PM_2.5_ species, e.g. cohort studies that commonly use annual or multi-year averaged pollution levels.

### 3.4. Temporal and spatial representativeness of long-term predictions of PM_2.5_ speciation

**Fig. 4.**
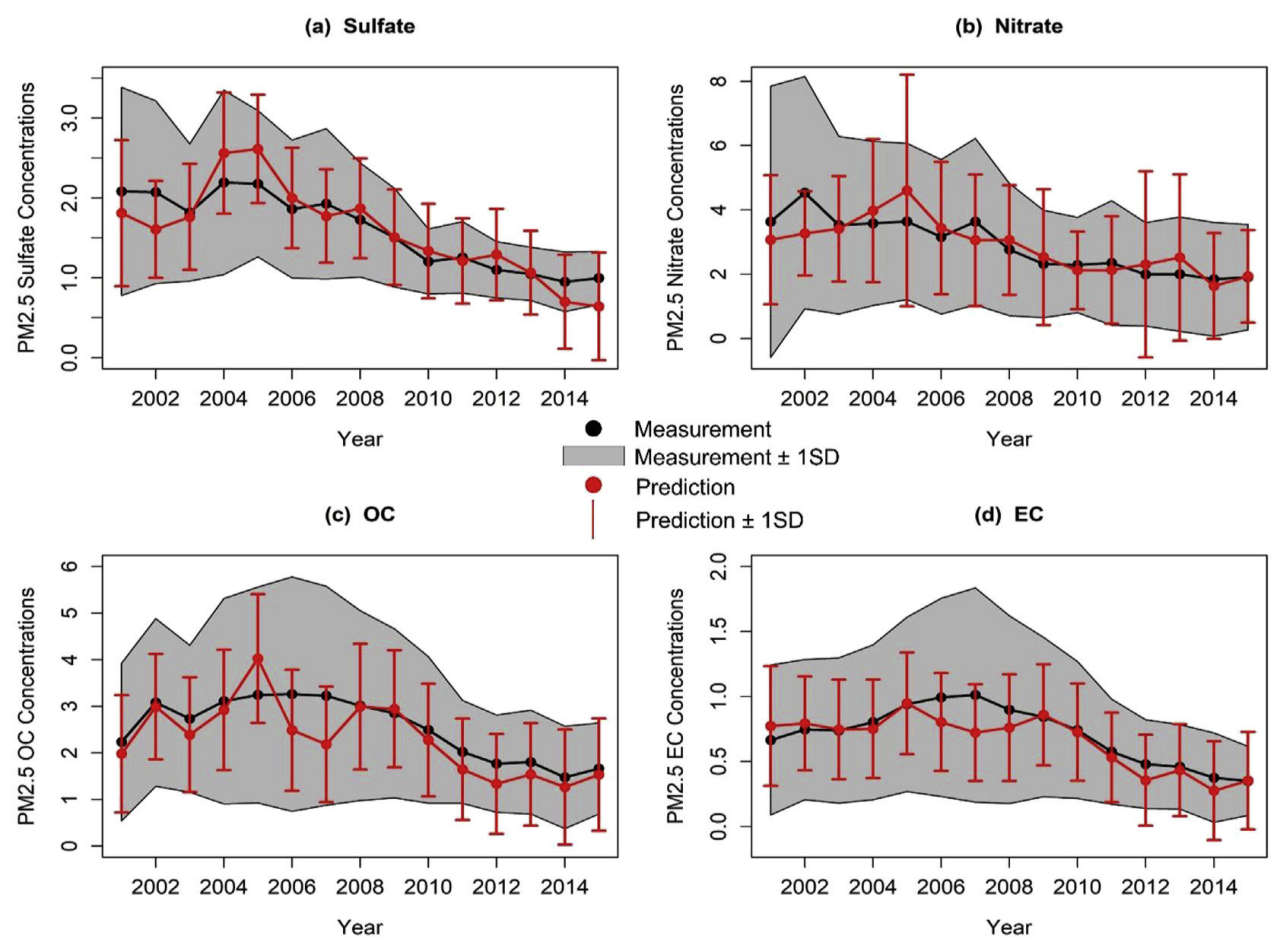
Temporal trends of averaged predicted annual concentrations of all MISR grids matched with monitoring sites and averaged measured annual concentrations of all PM_2.5_ speciation sites from 2001 to 2015. The predictions are from days when the corresponding MISR AOD values were available. The measurements are all available values of monitoring sites. Figures (a)–(d) denote the trends for PM_2.5_ sulfate, nitrate, OC and EC, respectively. X-axis represents year index, and y-axis represents annual mean concentrations of PM_2.5_ speciation with unit of μg/m^3^. Black and red dots represent measurement and prediction, respectively. The grey curves represents measurement ± 1 time standard deviation. The red bars represents prediction ± 1 time standard deviation. (For interpretation of the references to colour in this figure legend, the reader is referred to the Web version of this article.)

Evaluation of the accuracy of annual predictions of PM_2.5_ speciation needs to take into account the fact that the sampling frequency of MISR is every 16 days since only one path of MISR data is included in this study. Whether the annual prediction based on such sparse data can represent the true annual concentrations of PM_2.5_ speciation was analyzed in this study. For each MISR grid cell collocated with a ground monitor, measured annual concentrations based on all available daily measurements of the corresponding site and predicted annual concentrations on days with available MISR AOD values were calculated. The correlation coefficients between predicted and measured annual concentrations during the entire study period based on 202 pairs of points are 0.86, 0.78, 0.84 and 0.87 for PM_2.5_ sulfate, nitrate, OC and EC, respectively. The annual NME values are 23%, 32%, 29% and 29% for PM_2.5_ sulfate, nitrate, OC and EC, respectively, which are significantly better compared with the daily NME. This comparison shows that the predicted annual concentrations based on the MISR data are highly correlated with measured annual concentrations for PM_2.5_ sulfate, nitrate, OC and EC; and the representativeness of the predicted annual concentrations is promising. Next, we averaged annual concentrations from all MISR grid locations in the study area for predicted values and measured values separately to see if the long-term trends reflected by the predictions are similar with those contained in the measurements. Fig. 4 shows the temporal trends of averaged predicted annual concentrations of all matched MISR grids and averaged measured annual concentrations of all PM_2.5_ speciation sites from 2001 to 2015. Both predicted and measured data show decreasing of PM_2.5_ sulfate, nitrate, OC and EC during the study period, with smaller decrease after 2010. The predicted annual concentrations agree well with measured annual concentrations in most years, but fluctuate during 2005–2007 when the standard deviations of measured annual concentrations are relatively high, indicating that the measurements varied more significantly during 2005–2007 than other periods, which makes it difficult to capture the extra variabilities based on current predictors and fitted models. The comparison indicates that the annual predicted concentrations of PM_2.5_ sulfate, nitrate, OC and EC are able to reasonably reflect the true long-term trend of the four PM_2.5_ species in California, even though MISR AOD sampling occurs less frequently than the ground monitor measurements.

There are only up to 15 PM_2.5_ speciation monitoring sites per year in our modeling domain, making it difficult to validate the spatial pattern of predictions in those areas that do not have monitoring sites. However, there are many more monitoring sites in our study domain that measure total PM_2.5_ mass concentrations. We reconstructed PM_2.5_ mass concentration based on the predictions of PM_2.5_ speciation and compared the results with all available daily PM_2.5_ mass concentration measurements from IMPROVE and EPA PM_2.5_ sites to see if the spatial patterns were similar. According to the IMPROVE formula (Malm et al., 1994), PM_2.5_ mass can be reconstructed as the sum of ammonium sulfate, ammonium nitrate, organic material, elemental carbon and soil dust (Lowenthal and Kumar 2003). We reconstructed PM_2.5_ mass based on sulfate, nitrate, organic material and EC here by using the following equation since we did not predict concentrations of PM_2.5_ soil dust in this study.

(4) $${\text{PM}}_{2.5}\text{Mass}=1.375\times {\text{SO}}_{4}^{2-}+1.29\times {\text{NO}}_{3}^{-}+1.4\times \text{OC}+\text{EC}$$

Daily PM_2.5_ mass measurements are downloaded from EPA AQS (https://aqs.epa.gov/aqsweb/airdata/download_files.html) and from IMPROVE network (http://views.cira.colostate.edu/fed) for remote/rural sites during 2001–2015. In our study domain, soil dust accounts for approximately 5%–10% of total PM_2.5_ mass in the South Coast Air Basin (CARB, 2016), which is relatively small and stable. Soil dust fractions of measured PM_2.5_ mass in southern California counties along coast are quite similar (Hand et al., 2017), but are different from that in Kern county and most areas of San Bernardino county (Hand et al., 2017). Therefore, the comparison is done along the coastal areas including South Central Coast, South Coast and San Diego County air quality management districts based on designations of the California Air Resource Board (CARB) (https://www.arb.ca.gov/desig/desig.htm), where the excluded soil dust fraction is relative uniform.

**Fig. 5.**
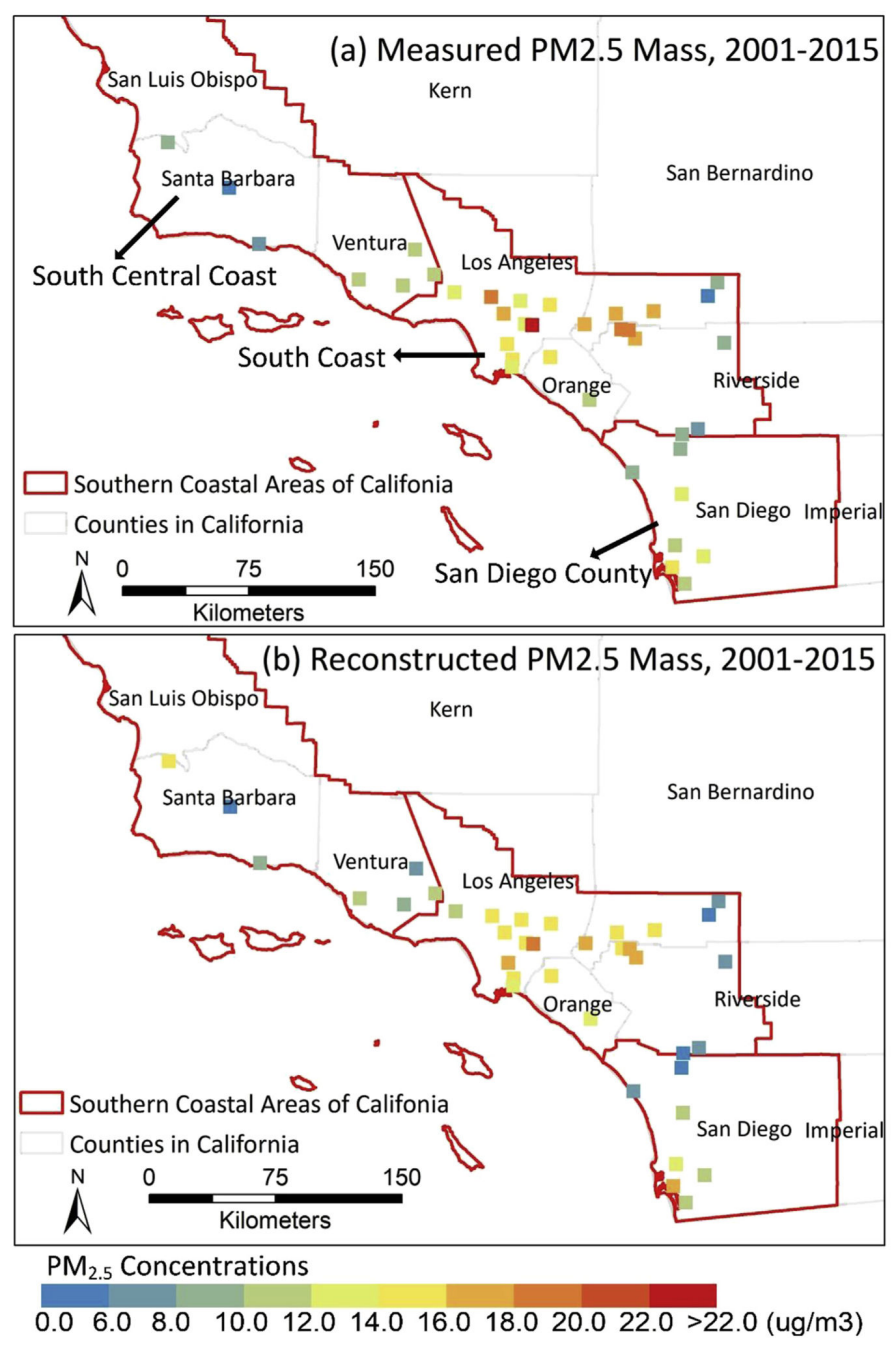
Comparison of 15-year averaged annual measured PM_2.5_ mass (a) and reconstructed PM_2.5_ mass in MISR grids (b) from 2001 to 2015 with unit of μg/m^3^. Grey outline represents the county boundary; red outline represents air quality management district designations of CARB. (For interpretation of the references to colour in this figure legend, the reader is referred to the Web version of this article.)

Comparison of 15-year averaged annual measured PM_2.5_ mass and reconstructed PM_2.5_ mass in MISR grids is displayed in Fig. 5, and comparisons based on 3-year averaged measured and predicted data are summarized in Figure S5 in the supplementary materials. The concentration of reconstructed PM_2.5_ mass is slightly lower than the gravimetric measurements since soil dust is not included. The spatial pattern of reconstructed PM_2.5_ mass, however, is highly consistent with that of measured PM_2.5_ mass. The reconstructed PM_2.5_ mass concentrations based on predicted PM_2.5_ components are able to illustrate the spatial variability of total PM_2.5_ mass and capture the hotspots in city clusters of Los Angeles, Orange, Riverside, San Bernardino and San Diego counties, as shown by measured PM_2.5_ mass concentrations. The temporal variation of reconstructed PM_2.5_ mass is also in line with the measured values, showing that the PM_2.5_ mass concentrations decreased gradually from 2001 to 2012 and slightly rebounded during 2013–2015, which was partly caused by extreme drought conditions in Southern California (CARB, 2016). The standard deviations of measured and reconstructed PM_2.5_ mass concentrations based on 15-year annual mean values in 2001–2015 in MISR grids are displayed in Figure S4. The spatial patterns of standard deviations are also similar for measured and reconstructed PM_2.5_ mass concentrations. Hence, these comparisons indirectly confirm that the spatial patterns of predicted PM_2.5_ speciation concentrations are reasonable.

### 3.5. GAMs based on MISR total AOD at 4.4-km resolution

We also developed GAMs for the four PM_2.5_ species based on daily measurements by using total AOD instead of fractional AODs and compared the model performances with those based on fractional AODs. The results of GAMs with MISR total AOD of 4.4-km resolution are summarized in Table S3. By comparing model performance in Table 1 and Table S3, our results show that the GAMs with fractional AODs have both moderately higher adjusted R^2^ and LOOCV R^2^ than those with total AOD for PM_2.5_ sulfate and nitrate; while the model performances with total AOD changes slightly for PM_2.5_ carbonaceous species comparing with those with fractional AODs. The results suggest that models with fractional AODs show improved capability in explaining the variability of PM_2.5_ species as compared with models with total AOD, especially for sulfate and nitrate.

### 3.6. GAMs based on MISR fractional AODs at 17.6-km resolution

In order to compare the predicted concentrations produced by current MISR aerosol products at 17.6-km resolution with the upcoming product at 4.4-km resolution, we also developed the GAMs following (equation 3) based on MISR fractional AODs at 17.6-km resolution in the same study domain and produced the prediction surfaces for PM_2.5_ sulfate, nitrate, OC and EC. Since this is a sensitivity analysis, we only processed the MISR 17.6-km data for 2007–2012. There are 180 matched site-days using MISR 17.6-km fractional AODs as compared to 302 site-days for MISR 4.4-km fractional AODs in the same study period. Results of model fitting and prediction maps of 2007–2012 are in Table S4 and Figure S4. Generally, the model performances with the two MISR aerosol products of different spatial resolution are comparable. Comparing to the results shown in Table 1, models with the 17.6-km fractional AODs performed slightly worse than those with 4.4-km fractional AODs for sulfate and nitrate, but slightly better than those with 4.4-km fractional AODs for OC and EC. As compared with the17.6-km prediction surface, the 4.4-km maps can capture higher variability and smaller spatial gradients of PM_2.5_ species’ concentrations, especially within urban areas; which is important to accurately assess exposure levels in epidemiological studies.

### 3.7. Limitations

Although the analysis suggests that the GAMs developed based on MISR fractional AODs can be used to predict the major chemical components of PM_2.5_ at a fine spatial scale and capture their spatial variabilities and long-term temporal trends, this study has several limitations. First, the relatively small model fitting datasets may limit the capacity of the models to select predictor variables for predicting PM_2.5_ species concentrations, subsequently to affect model stability and prediction accuracy. Model stability and prediction accuracy are expected to improve with additional MISR data from neighboring paths in the study domain as more high-resolution data become available in the near future. Second, our GAMs assume that the associations between MISR AODs and PM_2.5_ speciation concentrations are constant across the entire modeling domain and 15-year time period. Given our relatively small study domain, this assumption may not cause substantial prediction error. Third, although the spatial resolution of PM_2.5_ speciation prediction maps is as high as 4.4 km, it is not feasible to study daily PM_2.5_ speciation concentrations based on GAMs developed in this study due to MISR’s low sampling frequency and missing data due to cloud cover. However, the results show good agreements between annual predicted concentrations and annual observed concentrations of PM_2.5_ speciation. Hence, the annual predicted concentrations of PM_2.5_ speciation are able to capture long-term variations of PM_2.5_ speciation. In addition, we used column MISR fractional AODs as the main predictors for surface PM_2.5_ speciation concentrations without considering aerosol vertical distribution. Satellite-estimated PM_2.5_ concentrations have been proven to be well correlated with ground observations in a well-mixed boundary layer and under clear sky conditions (Hoff and Christopher, 2009); therefore, it is reasonable to predict ground-level aerosols with column AOD. On the other hand, the correlations of ground-level PM_2.5_ and AOD can be improved by considering the vertical profile of aerosols (Hoff and Christopher, 2009). Currently, chemical transport models such as GEOS-Chem can provide aerosol vertical structures with a coarse spatial resolution and relatively low accuracy, which might be not suitable in our analysis in a small study domain. Alternatively, we added PBLH as one of predictors in the models, which helped improve the relation between surface PM_2.5_ speciation concentrations and satellite AOD (Liu et al., 2005). Scaling MISR column fractional AOD to surface level might improve the relation between PM_2.5_ speciation concentrations and MISR fractional AODs might PM_2.5_be the future research direction, when aerosol vertical profiles with a high spatial resolution and accuracy become available. Finally, as is true for all statistical models, the GAMs developed in study are specifically tuned for the Southern CA domain, which may not perform as well if applied to other regions.

## 4. Conclusions

This analysis is the first attempt at producing prediction surfaces of PM_2.5_ sulfate, nitrate, OC and EC by using MISR 4.4 km-resolution prototype fractional AOD components at the sub-regional scale. Our predictions of PM_2.5_ speciation based on the current modeling strategy have higher accuracy compared to previous CTM simulation results, and have a finer spatial resolution compared to results based on MISR 17.6 km-resolution product. Our analysis suggests that this data product, coupled with the modeling approach described here, is able to explain the most variability of concentrations of major PM_2.5_ chemical components in Southern California. The 4.4 km-resolution MISR fractional AODs based predictions successfully capture hotspots and finer gradient variability of the four PM_2.5_ species within our study region. Distinguishing the high spatial variability of PM_2.5_ species is also crucial for exposure assessment in health studies. Despite the low daily sampling frequency of MISR aerosol product, the annual concentrations of PM_2.5_ species are well represented by the predicted annual concentrations based on the models developed in this study. The long-term mean predictions based on MISR fractional AODs provide a promising way to estimate concentrations of PM_2.5_ species, which will be valuable for understanding the effectiveness of air pollution control policy and supporting epidemiological studies by linking estimated PM_2.5_ speciation data with common spatial units such as census tract or zip code. Further predictions of PM_2.5_ speciation concentrations at the local to global scales will be possible when Version 23 MISR aerosol product is publicly released in the near future.

## Supplementary Material

Supplemental

## Figures and Tables

**Table 1:** Results of model fitting of PM_2.5_ sulfate, nitrate, OC and EC with MISR fractional AODs of 4.4-km resolution.

Species	Significant variables	Adjusted R^2^	LOOCV R^2^
SO_4_^2-^	AOD1, AOD3, AOD6, AOD19, (x,y), elevation, DOY, Year, PBLH	0.66	0.62
NO_3_^−^	AOD2, AOD3, AOD6, AOD19, (x,y), road length, DOY, Year, PBLH	0.62	0.56
OC	AOD2, AOD3, (x,y), road length, DOY, Year	0.55	0.49
EC	AOD2, AOD14, AOD19, (x,y), road length, DOY, Year, PBLH	0.58	0.53

**Table 2:** RMSE, means of APE and NME of daily measured and model predicted concentrations of PM_2.5_ sulfate, nitrate, OC and EC.

Species	RMSE (μg/m^3^)	APE (μg/m^3^)	NME
SO_4_^2-^	0.90	0.61	37%
NO_3_^−^	1.88	1.31	54%
OC	1.34	0.95	45%
EC	0.41	0.30	51%
