# Supplementary material for: Estimating PM2.5 speciation concentrations using prototype 4.4 km-resolution MISR aerosol properties over Southern California
Source: Atmos Environ (1994). Author manuscript; Available in PMC 2018 Dec 11. (PMC6288801; doi:10.1016/j.atmosenv.2018.03.019)
Supplement: Supplemental [file NIHMS989249-supplement-Supplemental.docx]

Estimating PM_2.5_ speciation concentrations using prototype 4.4 km-resolution MISR aerosol properties over Southern California

Xia Meng, Michael J. Garay, David J. Diner, Olga V. Kalashnikova, Jin Xu, Yang Liu

**Information about MISR fractional AOD components.**

The 74 mixtures are constructed from eight fractional AOD components (AOD1, AOD 2, AOD 3, AOD 6, AOD 8, AOD 14, AOD 19 and AOD 21 in the MISR Aerosol Physical and Optical Properties database) that represent different particle characteristics of size, shape and complex refractive index ([Liu et al., 2009](#_ENREF_2)). The eight fractional AOD components (listed in Table S1) are named starting with particle shape, followed by a qualitative scattering property designation and ending with the effective radius for a number-weighted log-normal distribution. Single scattering albedo at 558 nm wavelength is added when necessary to distinguish components.

Table S1. Aerosol components assumed in MISR Version 23 experimental retrievals.

| Fractional AOD | Aerosol components | Name | d_min_^1^ | d_max_^1^ | d_c_^1^ | Single scattering albedo (558nm) |
| --- | --- | --- | --- | --- | --- | --- |
| AOD1 | Particle1 | Spherical_nonabsorbing_0.06 | 0.002 | 0.8 | 0.06 | 1 |
| AOD2 | Particle 2 | Spherical_nonabsorbing_0.12 | 0.002 | 1.5 | 0.12 | 1 |
| AOD3 | Particle 3 | Spherical_nonabsorbing_0.26 | 0.02 | 3.0 | 0.24 | 1 |
| AOD6 | Particle 6 | Spherical_nonabsorbing_2.8 | 0.2 | 100 | 1.00 | 1 |
| AOD8 | Particle 8 | Spherical_absorbing_0.12_ssa_green_0.9 | 0.002 | 1.5 | 0.12 | 0.9 |
| AOD14 | Particle 14 | Spherical_absorbing_0.12_ssa_green_0.8 | 0.002 | 1.5 | 0.12 | 0.8 |
| AOD19 | Particle 19 | Grains_mode1_h1 (dust) | 0.2 | 2.0 | 1.00 | 0.98 |
| AOD21 | Particle 21 | spheroidal_mode2_h1 (dust) | 0.2 | 12.0 | 2.00 | 0.90 |

1. d_min_: the minimum particle diameters for a given aerosol component;

d_max_: the maxmum particle diameters for a given aerosol component;

d_c_: the characteristic diameter of the lognormal size distribution.

**Information about differences in the IMPROVE and CSN carbon measurements.**

The differences in the IMPROVE and CSN carbon measurements are mainly caused by different samplers and analytical methods. CSN used different sample methods from IMPROVE and began transitioning sampling on quartz-fiber filters for carbonaceous components from MetOne SASS and other samplers to the IMPROVE URG 3000N sampler in 2007([Solomon et al., 2014](#_ENREF_4)). EC and OC data from IMPROVE are measured by the Thermal Optical Reflectance (TOR) method; while these data from CSN were measured by the Thermal Optical Transmittance (TOT) method before 2007 and started to transition to the IMPROVE TOR method since 2007([Solomon et al., 2014](#_ENREF_4)). Previous analysis indicated that the conversion can improve the consistency of carbonaceous measurements from the CSN and IMPROVE networks ([Hand, 2011](#_ENREF_1)).If OC and EC from CSN were measured by TOT method, the EC data was adjusted by multiplying by 1.3 to match the EC data from IMPROVE, while the OC data was converted with the following equation ([Malm et al., 2011](#_ENREF_3)):

OC_adjust = ((OC_CSN_ – 0.3 × EC_CSN_) – A)/M

Table S2. A and M values in equation (2) for OC conversion.

| M (unitless) | 1.2 |
| --- | --- |
| A_Jan_ (µg/m^3^) | 1.1 |
| A_Feb_ (µg/m^3^) | 1.3 |
| A_Mar_ (µg/m^3^) | 1.2 |
| A_Apr_ (µg/m^3^) | 1.4 |
| A_May_ (µg/m^3^) | 1.6 |
| A_Jun_ (µg/m^3^) | 1.7 |
| A_Jul_ (µg/m^3^) | 1.8 |
| A_Aug_ (µg/m^3^) | 1.9 |
| A_Sep_ (µg/m^3^) | 1.5 |
| A_Oct_ (µg/m^3^) | 1.2 |
| A_Nov_ (µg/m^3^) | 1.0 |
| A_Dec_ (µg/m^3^) | 1.1 |

**Comparing results from CSN data and IMPROVE data.**

Table S3. Predicted errors of daily measured and model predicted concentrations of PM2.5 sulfate, nitrate, OC and EC based on CSN data and IMPROVE data, respectively.

| Species | CSN | |  | IMPROVE | |
| --- | --- | --- | --- | --- | --- |
|  | Daily Mean (µg/m^3^) | NME |  | Daily Mean  (µg/m^3^) | NME |
| SO_4_^2-^ | 2.28 | 34% |  | 1.27 | 41% |
| NO_3_^-^ | 3.38 | 46% |  | 1.89 | 62% |
| OC | 3.43 | 40% |  | 1.40 | 51% |
| EC | 0.99 | 39% |  | 0.38 | 66% |

**Results of model fitting for sensitive analysis.**

Table S3. Results of model fitting of PM_2.5_ sulfate, nitrate, OC and EC with MISR total AOD of 4.4-km resolution.

| Species | Significant variables | Adjusted R^2^ | LOOCV R^2^ |
| --- | --- | --- | --- |
| SO_4_^2-^ | AOD, (x,y), elevation, DOY, Year, PBLH | 0.61 | 0.58 |
| NO_3_^-^ | AOD, (x,y), road length, DOY, Year, PBLH | 0.47 | 0.44 |
| OC | AOD, (x,y), road length, DOY, Year | 0.54 | 0.51 |
| EC | AOD, (x,y), road length, DOY, Year | 0.56 | 0.53 |

Table S4. Results of model fitting of PM_2.5_ sulfate, nitrate, OC and EC with MISR fractional AODs of 17.6-km resolution.

| Species | Significant variables | Adjusted R^2^ | LOOCV R^2^ |
| --- | --- | --- | --- |
| SO_4_^2-^ | AOD1, AOD2, AOD8, AOD14, elevation, DOY, Year, PBLH | 0.61 | 0.50 |
| NO_3_^-^ | AOD6, AOD8, (x,y), road length, DOY, Year, PBLH | 0.60 | 0.49 |
| OC | AOD2, AOD8, (x,y), road length, DOY, Year, PBLH | 0.67 | 0.55 |
| EC | (x,y), road length, DOY, Year, PBLH | 0.65 | 0.60 |


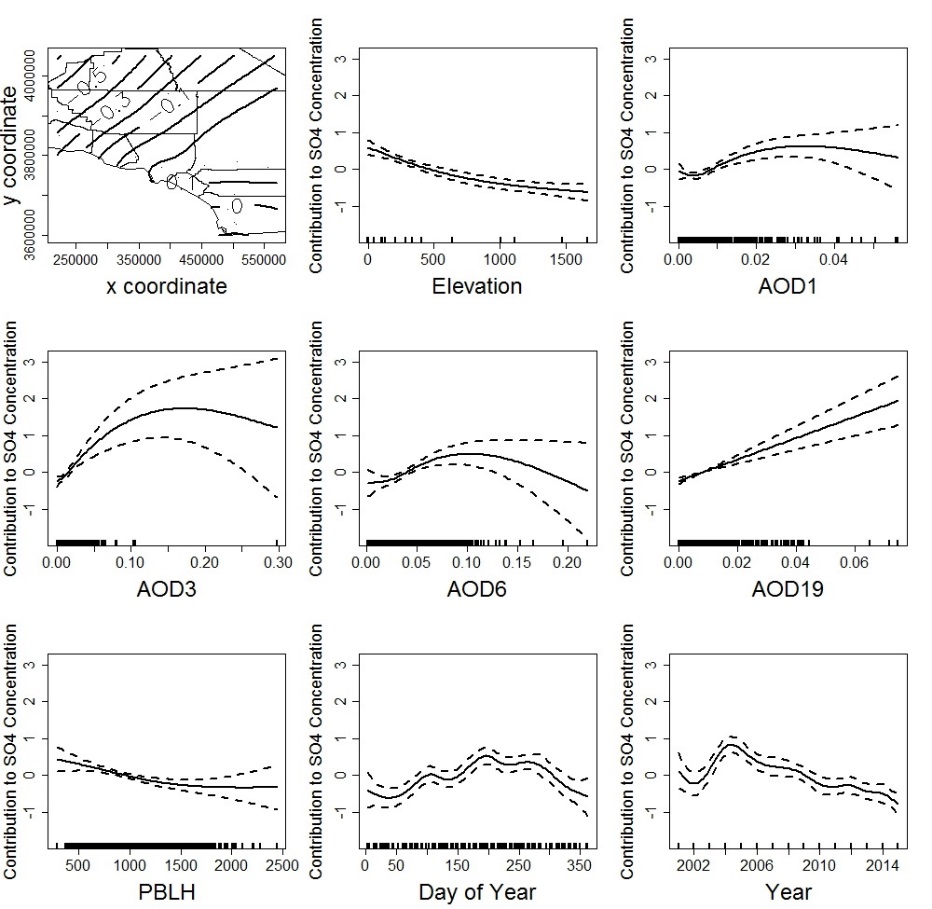

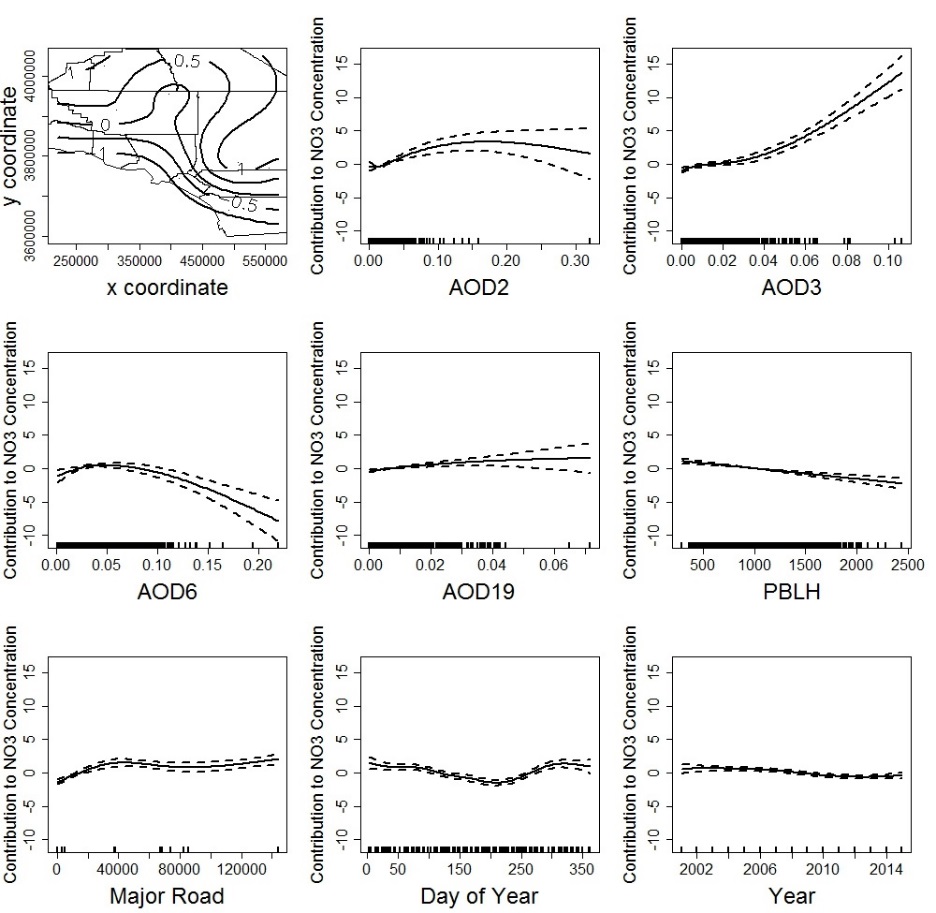
(a) Sulfate (b) Nitrate

(c) OC (d) EC


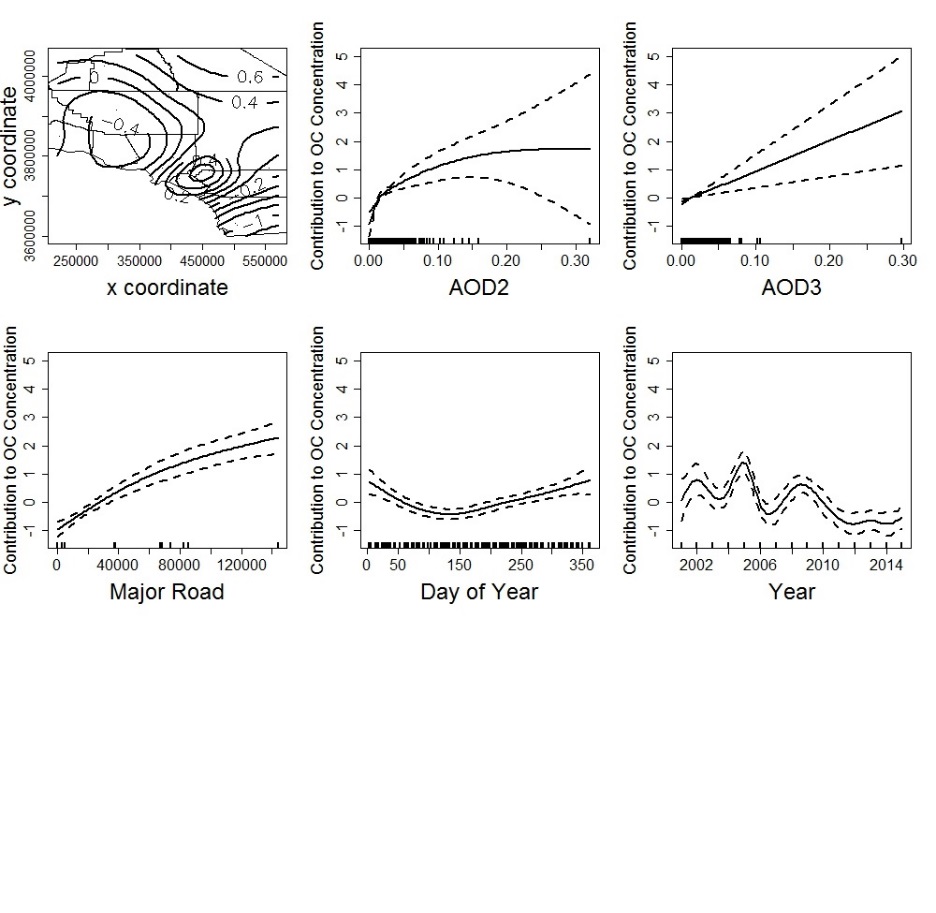

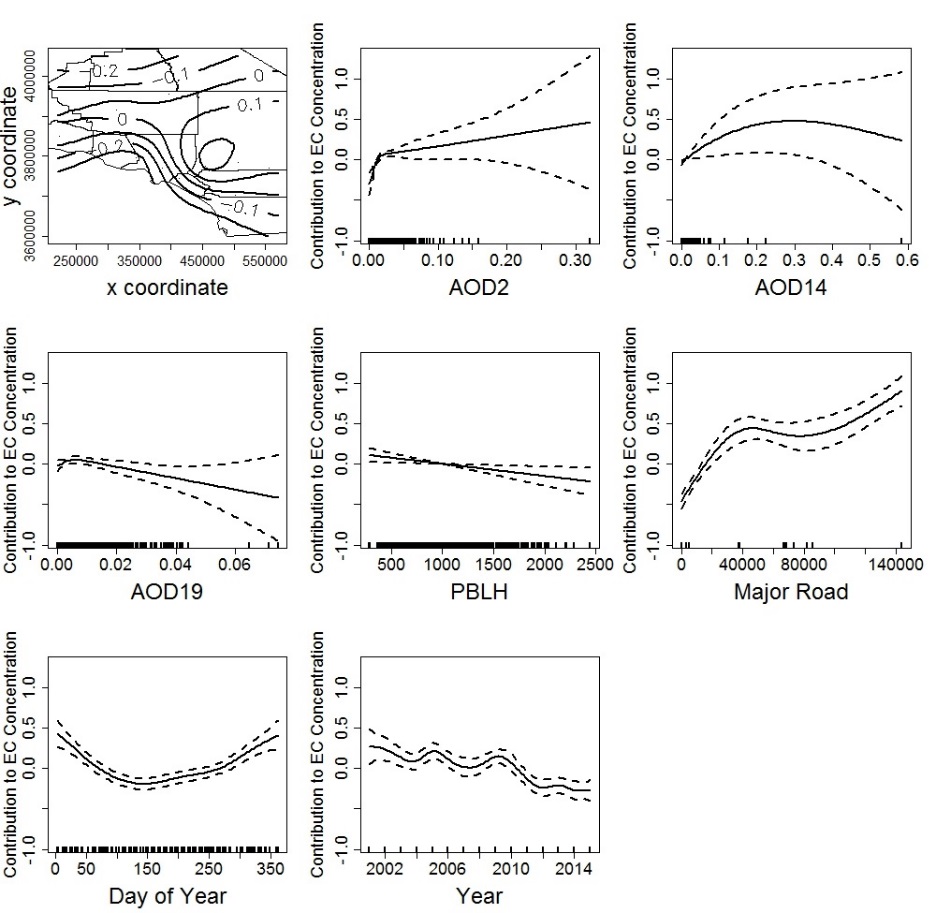


Figure S1. Smooth curves of predictor variables in GAMs for PM_2.5_ sulfate (a), nitrate (b), OC (c) and EC (d).

1. Locations of ports in Southern California (http://www.worldportsource.com/ports/USA_CA.php)


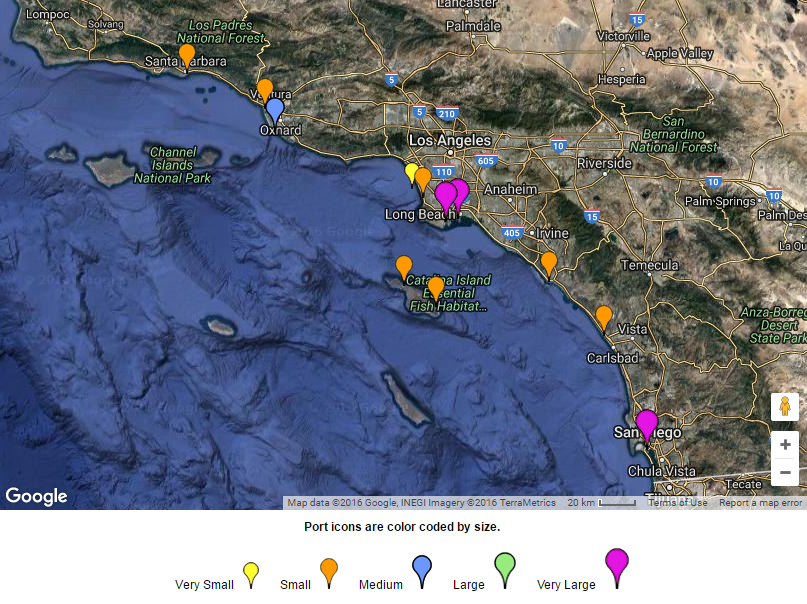


1.
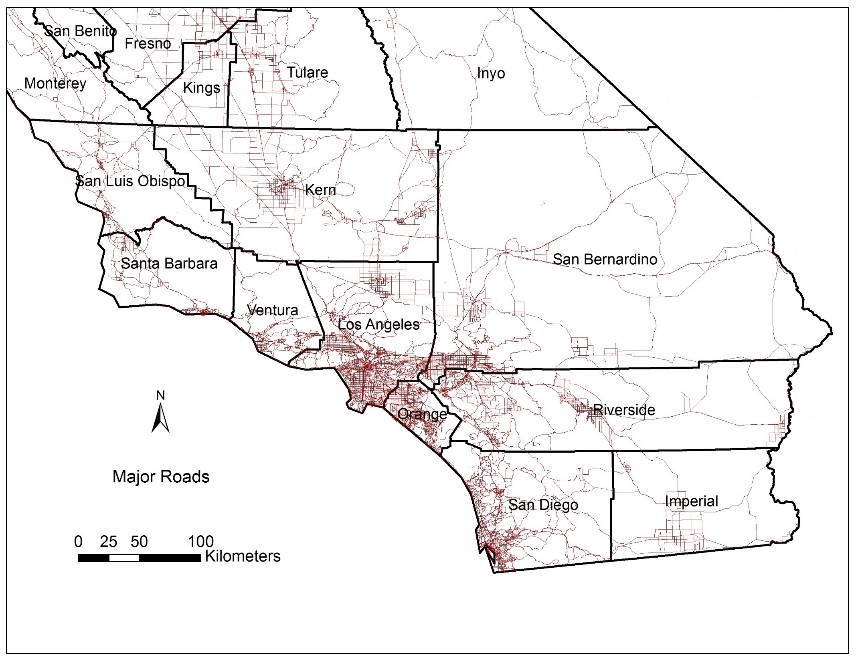
Map of major roads in Southern California
2. Distribution of city light in Southern California


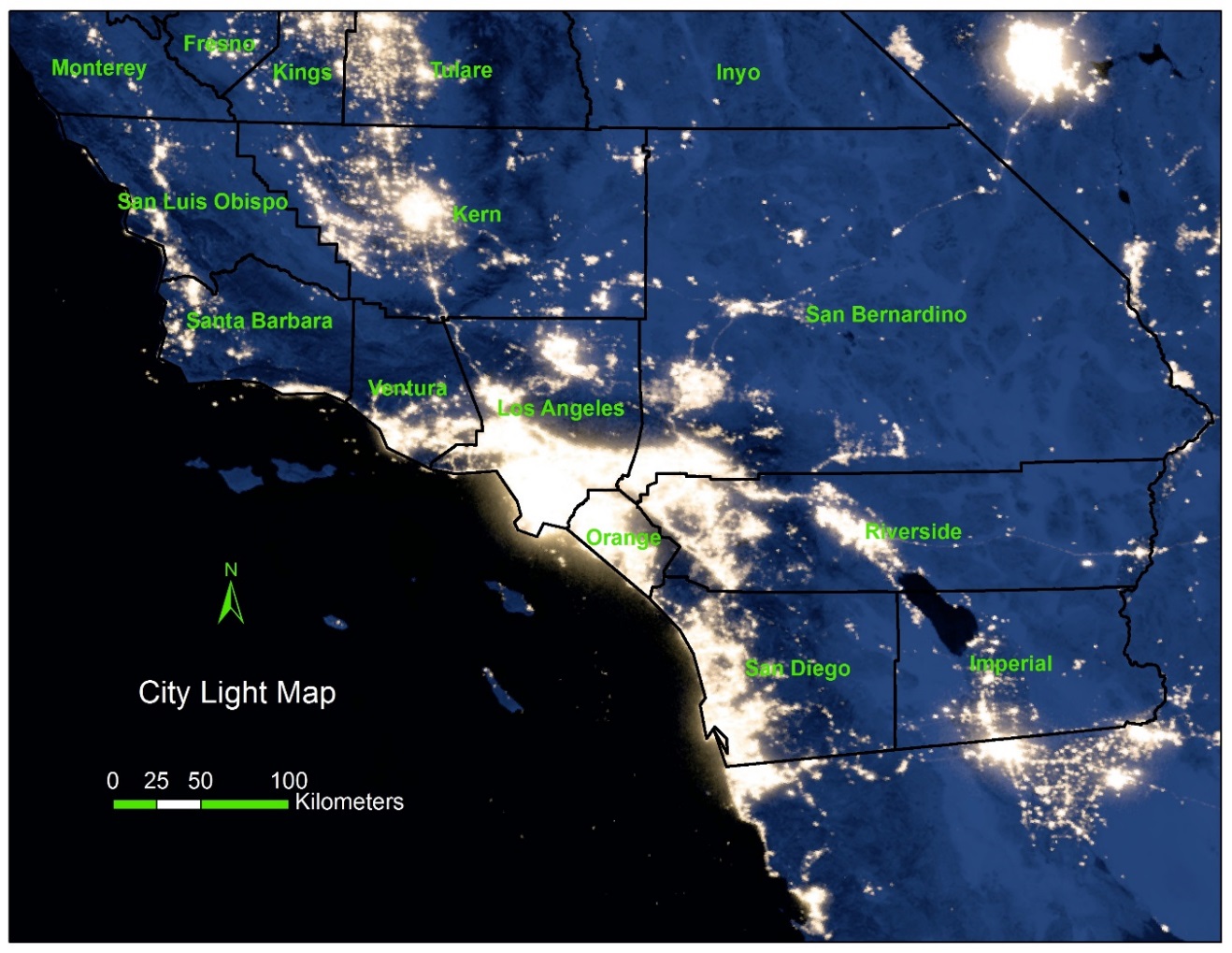


Figure S2. The locations of ports, map of major roads and distribution of city lights from NASA as indicator of cities and population. These figures are listed to help readers understand the spatial characteristics of PM_2.5_ species in figure 3. For example, areas along the coastline with seaports have the highest PM_2.5_ sulfate concentration, particularly in Los Angeles area; the hotspots of PM_2.5_ carbonaceous components appear in urban areas and along major roads, showing the potential influence of anthropogenic combustion and traffic emissions


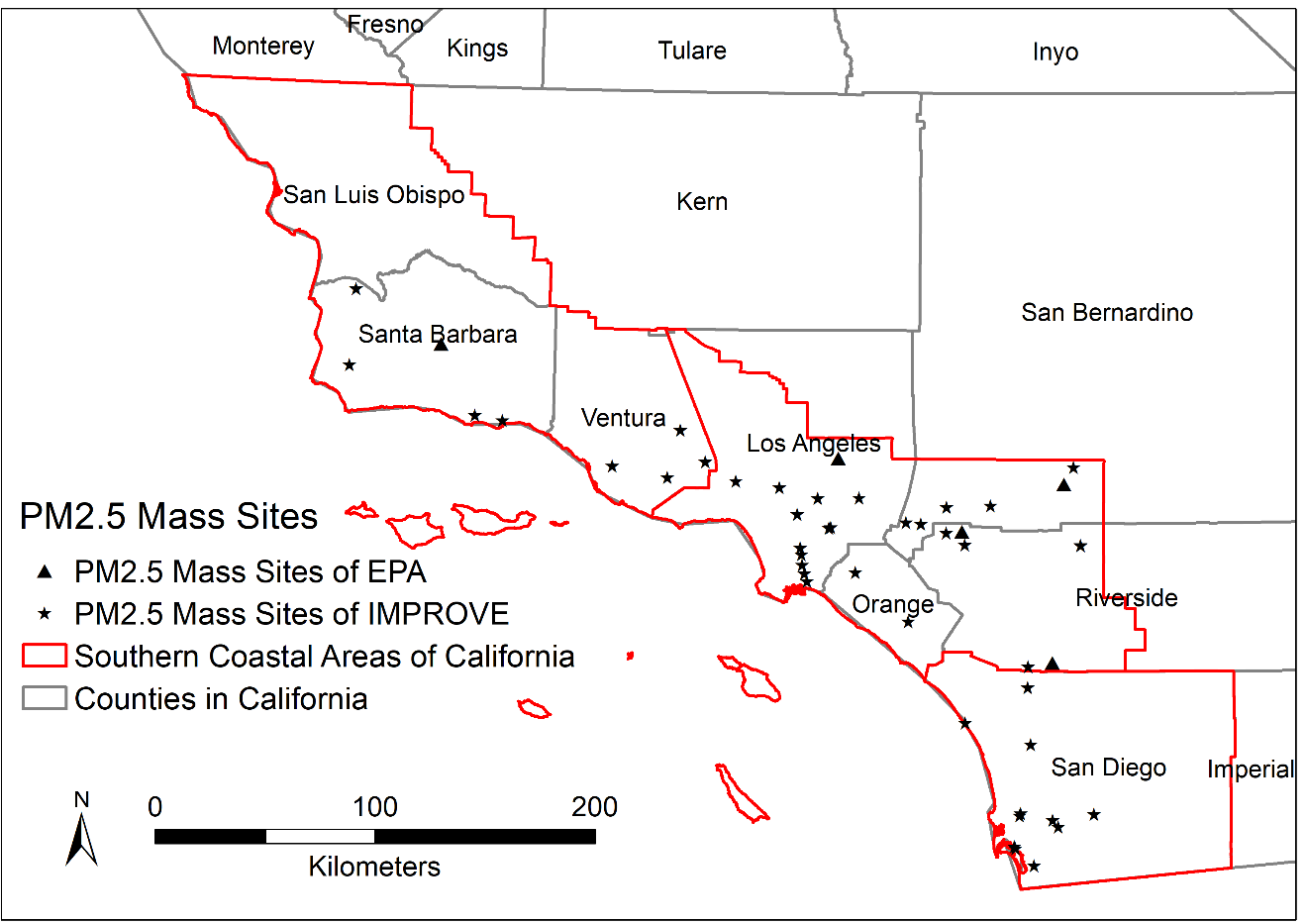


Figure S3 Locations of PM2.5 mass sites from EPA and IMPROVE networks in Southern Coastal Areas of California included in sensitive analysis. Southern Coastal Areas of California includes Southern Central Coast, Southern Coast, and San Diego County of CARB management districts from north to south.


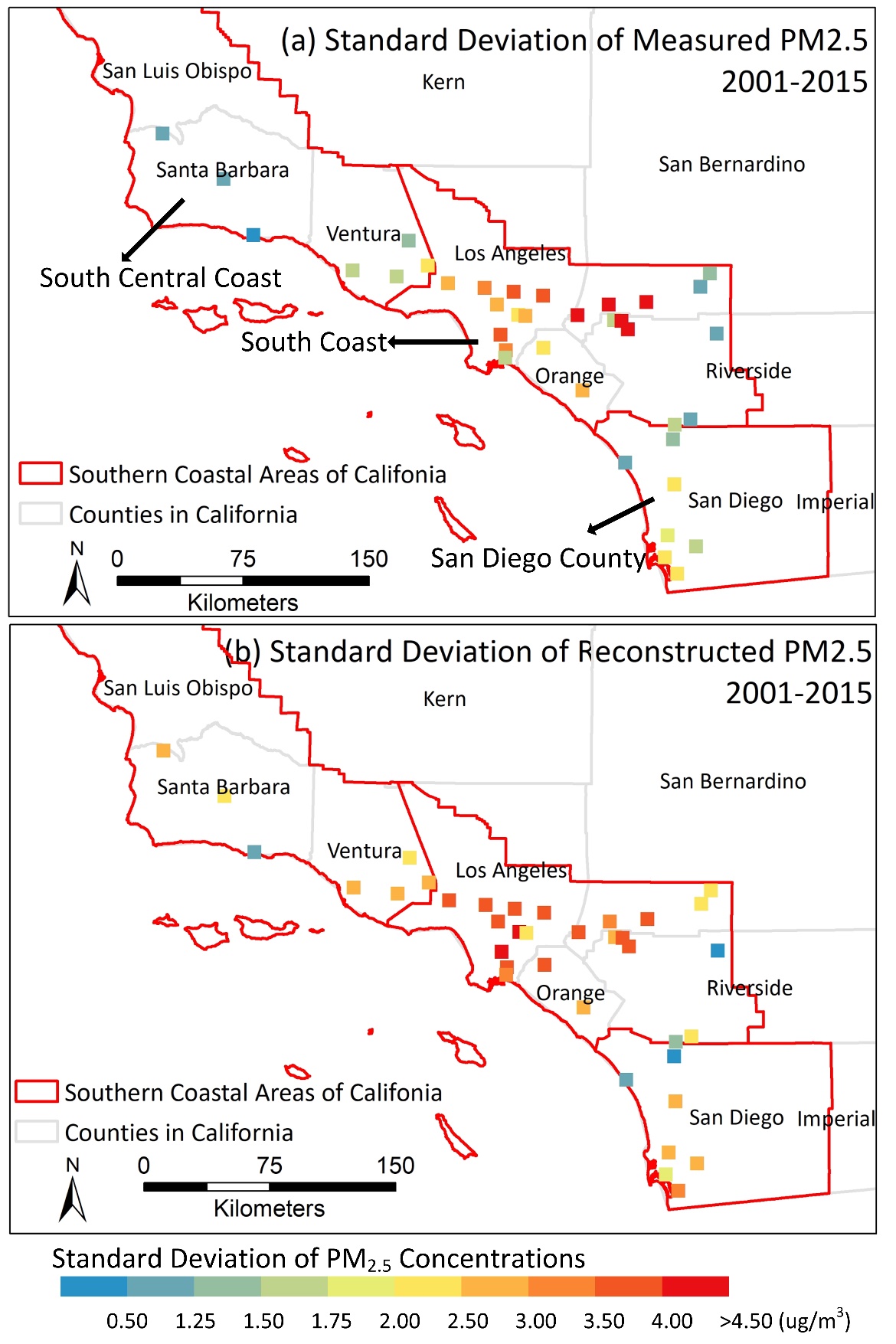


Figure S4. Spatial distribution maps of standard deviations of measured and reconstructed PM2.5 mass concentrations in MISR grids based on annual mean data in 2001-2015.


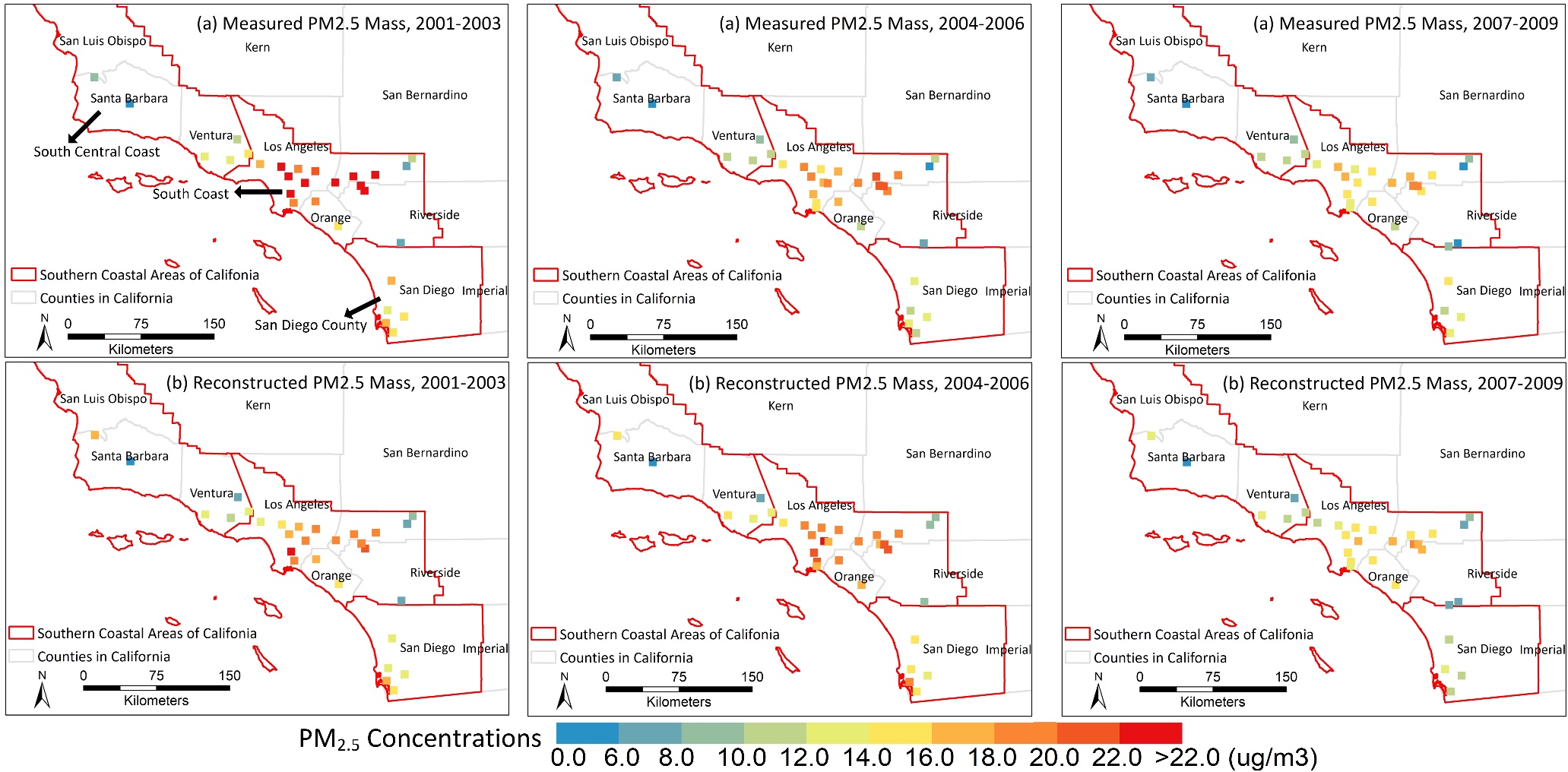


(Figure S5 continues in next page)


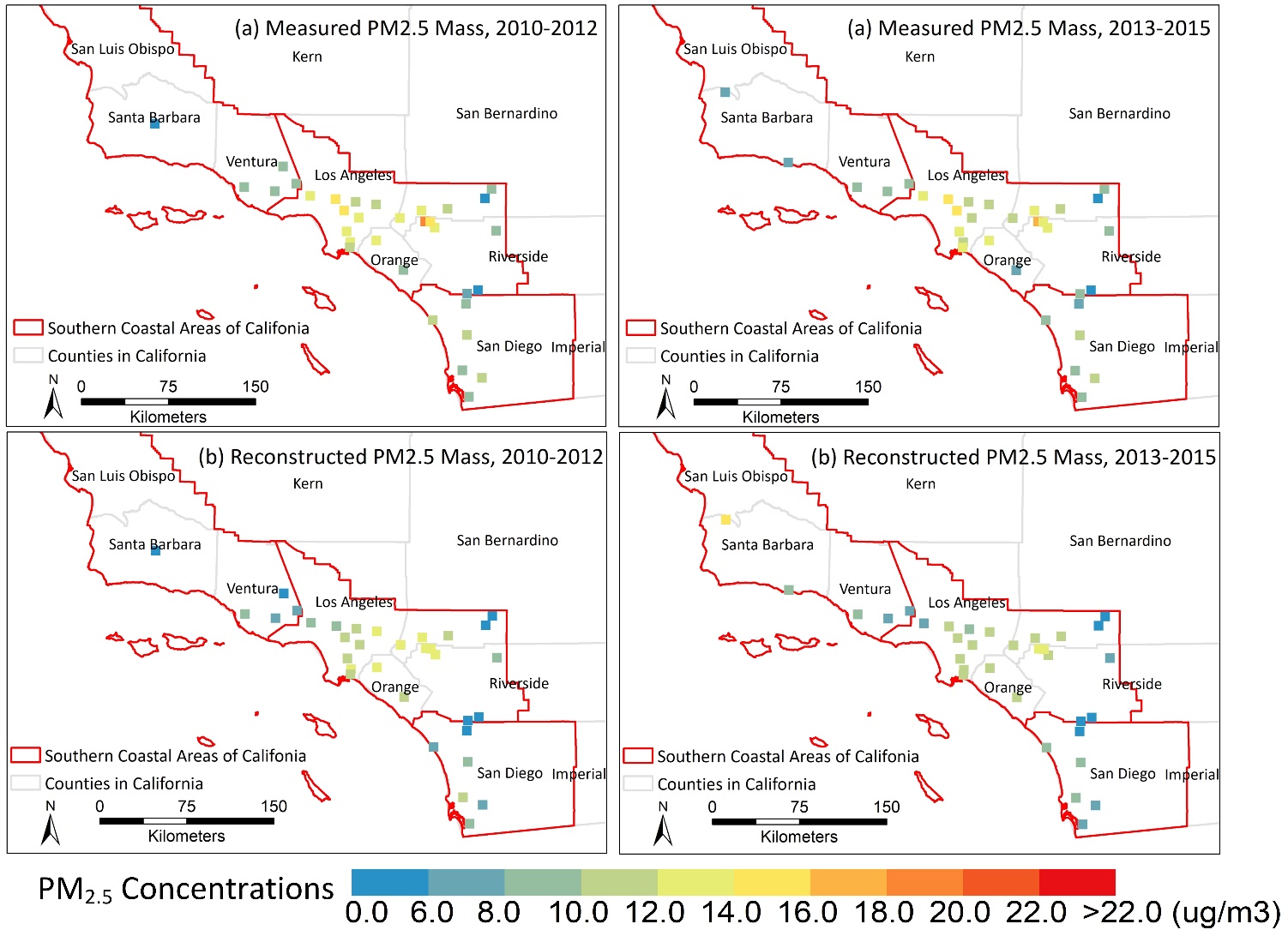


Figure S5. Comparisons between 3-year averaged annual concentrations of PM_2.5_ mass measurements and 3-year averaged annual concentrations of reconstructed PM_2.5_ mass based on predictions of PM_2.5_ speciation.


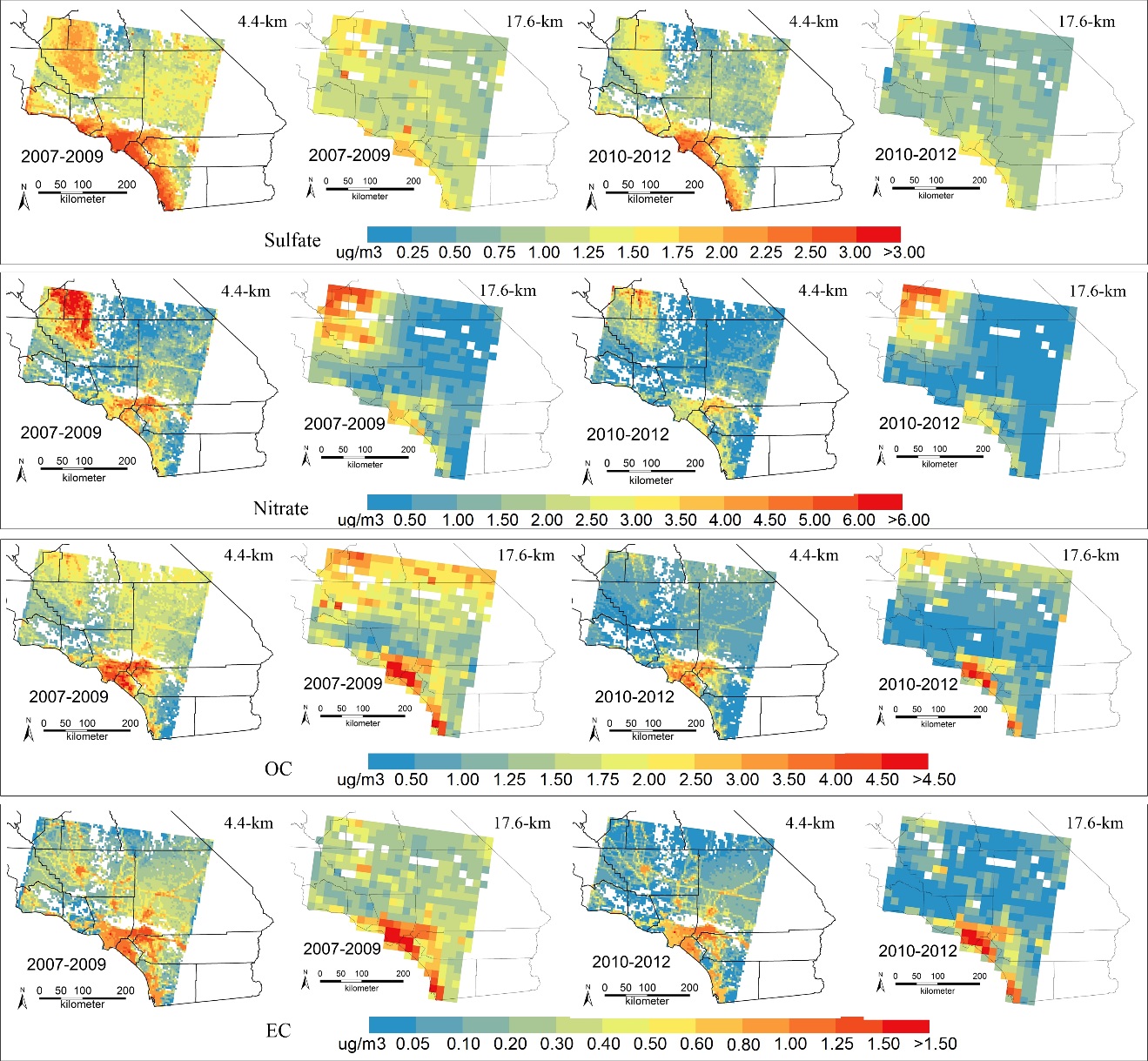


Figure S6. Comparison of prediction surfaces of 3-year averaged annual mean concentrations of PM_2.5_ sulfate, nitrate, OC and EC based on available MISR AOD data of 4.4-km resolution and available MISR AOD data of 17.6-km resolution, respectively. The prediction maps of years 2007-2009 for 4.4 km and 17.6 km resolution are in the first two columns, respectively, followed by the prediction maps of years 2010-2012 in the last two columns for each resolution, respectively.

**References**

Hand, J.L., Copeland, S. A., Day, D.E., Dillner A.N., Indresand, H., Malm, W.C., McDade, C.E., Moore, C. T., Pitchford, M.L., Schichtel, B. A., Watson, J. G., 2011. Spatial and Seasonal Patterns and Temporal Variability of Haze and its Constituents in the United States: Report V June 2011. IMPROVE Reports.

Liu, Y., Schichtel, B.A., Koutrakis, P., 2009. Estimating Particle Sulfate Concentrations Using MISR Retrieved Aerosol Properties. Ieee J-Stars 2, 176-184.

Malm, W.C., Schichtel, B.A., Pitchford, M.L., 2011. Uncertainties in PM2.5 Gravimetric and Speciation Measurements and What We Can Learn from Them. J Air Waste Manage 61, 1131-1149.

Solomon, P.A., Crumpler, D., Flanagan, J.B., Jayanty, R.K.M., Rickman, E.E., McDade, C.E., 2014. US National PM2.5 Chemical Speciation Monitoring Networks-CSN and IMPROVE: Description of networks. J Air Waste Manage 64, 1410-1438.
